# Causal Effects of Gut Microbiota and Associated Metabolites on Retinal Diseases and Visual Impairment: A Mendelian Randomization Study

**DOI:** 10.1155/joph/4233490

**Published:** 2026-06-17

**Authors:** Chuyao Yu, Li Dong, Ruiheng Zhang, Heyan Li, Xuhan Shi, Haotian Wu, Wenda Zhou, Yitong Li, Wen-Bin Wei

**Affiliations:** ^1^ Beijing Tongren Eye Center, Medical Artificial Intelligence Research and Verification Key Laboratory of the Ministry of Industry and Information Technology, Beijing Tongren Hospital, Capital Medical University, Beijing, 100730, China, ccmu.edu.cn

**Keywords:** causality, gut microbiota, gut–retina axis, Mendelian randomization, metabolites, retinal diseases, visual impairment

## Abstract

**Background:**

Previous observational study findings have indicated a vital association between gut microbiota features and retinal diseases based on the “gut–retina” axis. However, whether their relationships underlie causal effects remains to be established.

**Methods:**

Instrumental variables of 211 gut microbiota taxa were obtained from a genome‐wide association study (GWAS), and 28 gut‐associated metabolites and pathways were included as exposures. A two‐sample Mendelian randomization (MR) study was carried out to estimate gut microbiota effects on diabetic retinopathy (DR), early age‐related macular degeneration (eAMD), retinal detachments and breaks (RDs/RBs), retinal vascular occlusion (RVO), disorders of the choroid and retina (D‐C/R), and visual impairment. MR methods, including inverse variance weighted (IVW), MR‒Egger, weighted median, simple mode, and weighted mode methods, were used to investigate the causal relationship between gut microbiota features and various outcomes. Heterogeneity, pleiotropy, and stability tests of MR results were performed, and Bonferroni’s correction was used to test the strength of the causal relationships between exposures and outcomes, as well as reverse and multivariable MR analyses.

**Results:**

Through MR analysis of 211 microbes and six clinical phenotypes, a total of 35 gut microbiome and 3 associated metabolites were found to be associated with various outcomes. Cochrane’s *Q* test revealed that there was no significant heterogeneity between various single‐nucleotide polymorphisms. In addition, no significant level of pleiotropy was found according to the MR‒Egger and MR‐PRESSO global tests. After the Bonferroni‐corrected test, Genus *id.2041* (OR = 0.874, 95% CI: 0.816–0.936, *p* = 1.10e − 04, IVW) showed robust causality with D‐C/R, which had a nominal association with multiple other retinal diseases as well. Seven exposure–outcome effects markedly remained valid when BMI or alcohol intake frequency was separately included in multivariable MR analyses. According to the results of reverse MR analysis, no significant causal effect of outcomes was found on gut microbiota. No significant heterogeneity of instrumental variables or horizontal pleiotropy was found.

**Conclusion:**

We confirmed a potential causal relationship between some gut microbiota features and retinal diseases, thus providing new insights into the gut microbiota–mediated mechanism of retinopathy and indicating vital biomarkers for potential diagnostic, therapeutic, and prevention strategies.

## 1. Background

Retinal disease is a global health concern and affects billions of patients worldwide. Globally, it is estimated that 43.3 million people were blind in 2020; the number of people of all ages who are visually impaired is predicted to be approximately 834 million by 2050, 61 million of whom will suffer from vision loss [1]. Among retinal diseases, diabetic retinopathy (DR) is a frequent microvascular complication of diabetes [2], and approximately 75% of people with diabetes will develop DR 10 years after diagnosis [3]. Age‐related macular degeneration (AMD) is characterized by the accumulation of drusen in the macula, and it has been estimated that approximately 288 million people will be affected by AMD worldwide by 2040 [4]. Associated with ocular trauma [5], inflammation [6], and pathological myopia [7], retinal detachments and breaks (RDs/RBs) have drawn public attention for the prevention of their persistence and recurrence [8]. The estimated incidence of retinal vascular occlusion (RVO) has been slightly increasing in clinical observations since the COVID‐19 pandemic [9, 10]. With the progression of retinal diseases, persistent disorders of the choroid and retina (D‐C/R) have almost become inevitable, and visual impairment is not only a devastating development for affected individuals but also a major socioeconomic burden and family healthcare expenditure burden [11, 12]. Early identification and diagnosis and improvements in retinal disease medical interventions and prognoses are greatly important in current medical services.

In recent years, several new therapies have been developed to decrease the occurrence and progression of retinal diseases. Among them, hindering the progression of retinal diseases by intervening in the “gut–retina” axis is becoming a new research trend [13–16]. Gut microbiota, as the microorganisms regulating metabolism in the host intestine, also play an important role in the regulation of local and systemic immunity of the host [17, 18]. Emerging evidence shows that patients with retinal diseases are likely to have a certain degree of intestinal flora disorder [19], and microbiota‐targeting strategies have become potential preventive and therapeutic tools for retinal diseases. *Proteobacteria*, *Actinobacteria*, and *Firmicutes* have been shown to represent over 87% of all microorganisms present in the eye [20]. Metabolomics provides information on normal and pathological conditions, as well as on the effect and response to external stimuli [21, 22], and it is becoming an increasingly important tool in ophthalmic studies. Microbiota metabolomic profiles can be detected and measured from easily accessible biofluids or tissues [23], and the strength of association with disease outcomes also tends to be higher in metabolomics than in genetics. In contrast to observational studies, randomized controlled trials of gut microbiota abundance and associated metabolites could potentially help establish a relatively causal relationship. Unfortunately, owing to the influence of objective factors, such as technology and research methods, the screening of strains involved in early diagnosis and prognosis still has great limitations and bias. Therefore, whether the associations between gut microbiota and retinal diseases are causal and the direction of the causal associations is still unknown. More research is needed to validate whether gut microbiota abundance and associated metabolites affect retinal diseases independent of numerous confounding factors.

Mendelian randomization (MR) is an emerging and advanced epidemiological approach that mitigates many of the biases of observational studies or randomized controlled trials by using genetic variants as instrumental variables (IVs). MR eliminates the limitations of confounders, reverse causality issues, medical ethics, and high costs [24], providing a new and effective method for clinical research. Under specific assumptions, MR has the potential to evaluate potential causal effects between exposures (gut microbiota features) and a range of multiple outcomes (retinal diseases and visual impairment). This is consistent with the view that genes play a nonnegligible role in determining gut microbiome composition, making MR a valuable tool to assess the potential causal role of the “gut–retina” axis in human retinal diseases.

Our two‐sample MR study aimed to investigate the causal relationship among specified gut microbiota features (211 gut microbiota and 28 associated metabolites as exposures), retinal diseases (including DR, early age‐related macular degeneration [eAMD], RDs/RBs, RVO, and D‐C/R as outcomes), and visual impairment (as an outcome), which may provide guidance for developing helpful biomarkers for noninvasive diagnosis and potential therapeutic targets for retinal diseases.

## 2. Materials and Methods

### 2.1. Study Overview

**FIGURE 1:**
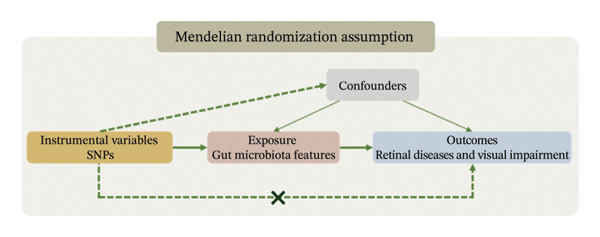
Overview of MR assumptions in this study.

**FIGURE 2:**
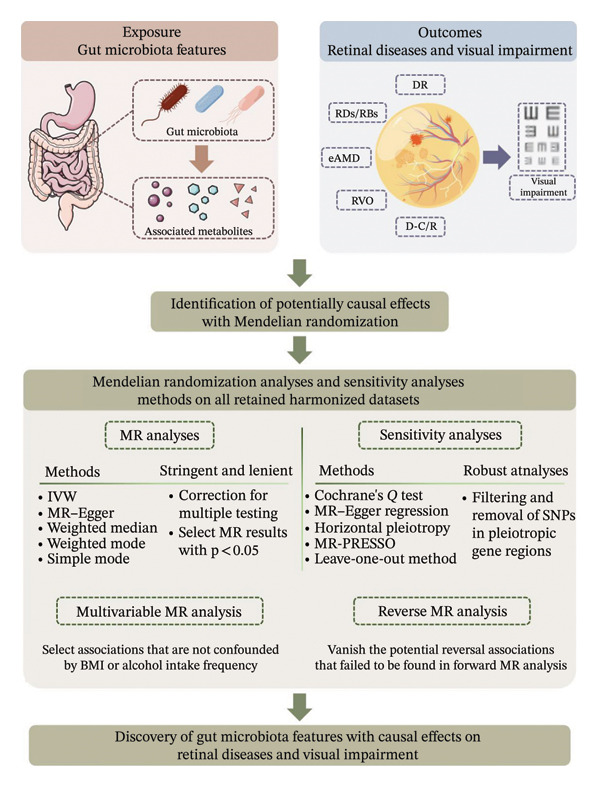
Overview of the MR framework used to investigate the causal effect.

To investigate potential causal links between gut microbial communities and retinal pathologies, we employed a two‐sample MR design. Genetic association summary statistics data from genome‐wide association study (GWAS) were obtained for retinal diseases [25]. Our analytical approach adhered to three core MR assumptions (Figure 1): First, selected genetic instruments must exhibit strong association with the exposure traits (gut microbiota features); second, these instruments should not be linked to known confounders of the exposure–outcome relationship; and third, there was no horizontal pleiotropy: IVs affected multiple outcomes only through gut microbiota features. The study design and reporting follow the STROBE‐MR guidelines [26]. An overview of the MR framework is displayed in Figure 2.

### 2.2. Data Source for Exposures and Outcomes

This study included a total of 211 gut microbiota (spanning 131 genera, 35 families, 20 orders, 16 classes, and 9 phyla) and 28 gut‐associated metabolites as exposure factors. Retinal disease outcomes were defined as follows: DR (a complication of diabetes mellitus affecting retinal vessels), eAMD (age‐related loss of vision in the macula, secondary to early‐stage retinal degeneration), RDs/RBs (the tears and separations of the inner retina layers from the underlying pigmented epithelium or neuroepithelium), and RVO (the blockage of retinal vascular circulation). To better reflect the multiple and prospective impacts of intestinal flora on retinal diseases and visual impairment, we further included two additional endpoints from cross‐sectional data from cohort studies: D‐C/R (the general term for several chorioretinopathies) and visual impairment (overall uncorrectable vision loss or impairment).

#### 2.2.1. Exposure Sources for Gut Microbiota Abundances and Associated Metabolites

Based on the MiBioGen consortium (https://www.mibiogen.org) [27], the full set of GWAS data for the gut microbiome was derived primarily from a large‐scale multiethnic GWAS meta‐analysis which included 18,340 individuals from 24 cohorts across 11 countries that recorded 211 gut microbiota taxa from the genus to phylum levels. Another aim was to explore whether 28 blood metabolites associated with the gut microbiota as well as their functional pathways could affect risk factors for retinal diseases and visual impairment. The genetic instruments for this analysis were selected from a GWAS of 1091 blood metabolites, based on data from 8299 individuals of European ancestry in the Canadian Longitudinal Study on Aging (CLSA) cohort (Supporting Table 2) [28].

#### 2.2.2. Outcomes Sources for Retinal Diseases

In order to identify genetic factors associated with eAMD, we confirmed the summary data for instrument variables derived from a GWAS meta‐analysis (European ancestry cohorts), with 14,034 cases and 91,214 controls. The data were sourced from 11 different datasets, including the International AMD Genomics Consortium (IAMDGC) and the UK Biobank (UKBB) [29]. In addition, the GWAS summary data for DR, RDs/RBs, RVO, D‐C/R, and visual impairment were from the European ancestry cohorts of the FinnGen consortium.

### 2.3. Selection Criteria for Instrumental Genetic Variants

We followed the methods of Ning Li et al. 2023 [30]. Selection criteria were as follows: (1) Single‐nucleotide polymorphisms (SNPs) associated with exposures at genome‐wide suggestive significance (*p* < 1 × 10^−5^ for microbiota; *p* < 5 × 10^−6^ for metabolites) [31, 32] were selected; (2) IVs with F‐statistic values (F = beta^2^/se^2^) < 10 were excluded; (3) independence among SNPs was achieved by applying a linkage disequilibrium (LD) threshold of *R* ^2^ < 0.001 within a 10,000 kb genomic window; (4) additional quality control steps were included removing SNPs with minor allele frequency (MAF) < 1%; (5) and excluding palindromic variants to prevent strand ambiguity and ensure accurate effect allele alignment. PhenoScanner [33] was used to identify potential confounding factors, ensuring that major microbiome‐related variables did not interfere with the relationship between exposure and outcomes. Missing data in exposures and covariates were addressed using multiple imputation. Imputed datasets were generated based on the observed data structure, and estimates were pooled according to Rubin’s rules to appropriately account for imputation uncertainty. To thoroughly assess the causal association of gut microbiota with retinal diseases and visual function, reverse causality MR was also conducted to eliminate biases. The selection of IVs described above ensured the robustness and reliability of our research findings.

### 2.4. MR Analysis

According to the methods of Kangcheng Liu et al. 2022 [34], inverse variance weighted (IVW), MR‒Egger, weighted median, weighted mode, and simple mode methods were used to investigate the causal relationship between exposure factors and outcomes. The IVW approach, serving as our primary analysis, combines Wald ratio estimates from individual SNPs through meta‐analysis, providing efficient causal estimates under the assumption of balanced pleiotropy. MR–Egger regression [35] is based on the assumption of InSIDE and mainly reflects the dose relationship between IVs and outcomes, taking into account a certain level of pleiotropy to remain valid. The weighted median method [36] provides consistent causal estimates even when up to 50% of the genetic instruments are invalid. In case of discrepancy across methods, IVW results were considered principal, following established MR conventions. We harmonized the summary statistics, removed SNPs with ambiguous strands, and aligned the data accordingly so as to ensure that each IV corresponded to the same effect allele. To control the potential false‐positive findings arising from multiple comparisons across all exposure–outcome tests, we applied a global correction using the Benjamini–Hochberg procedure and calculated the false discovery rate (FDR) q‐value, according to the number of bacteria under each attribute (genus: 0.05/131 (3.81 × 10^−4^), family: 0.05/35 (1.4 × 10^−3^), order: 0.05/20 (2.5 × 10^−3^), class: 0.05/16 (3.1 × 10^−3^), and phylum: 0.05/9 (5.5 × 10^−3^). All statistical analyses were performed using R software Version 4.2.3 (https://www.rproject.org/). The R package “TwoSampleMR” was used to perform MR analyses of the causal relationship between gut microbiota features and retinal diseases and visual impairment [37, 38].

### 2.5. Sensitivity Analysis

Several sensitivity analyses based on summary data via multiple IVs were conducted, as described below [24]. (1) Cochrane’s *Q* test was applied to quantify the heterogeneity of the IVs, with *p* < 0.05 considered heterogeneous; (2) the intercept of MR–Egger regression was used to assess the presence of potential pleiotropy in IVs, and horizontal pleiotropy was deemed to be nonexistent if *p* > 0.05; (3) multipotency was further analyzed using the MR‐PRESSO test to ensure the accuracy of results for gut microbiota taxa causally related to outcomes (based on IVW results), and possible outliers were removed; and (4) the leave‐one‐out method [38] was performed by omitting each instrumental SNP in turn to identify potential heterogeneous SNPs to further validate data robustness. We performed reverse MR analysis on the bacteria that were found to be causally associated with those outcomes in forward MR analysis. The methods and settings adopted were consistent with those of forward MR analysis. Multivariable MR analyses were conducted to determine whether the causal effects were robust to the inclusion of obesity and alcohol intake frequency. IVs with an F‐statistic less than 10 were considered potentially weak and were excluded from the analysis eventually to mitigate weak instrument bias.

## 3. Results

**FIGURE 3:**
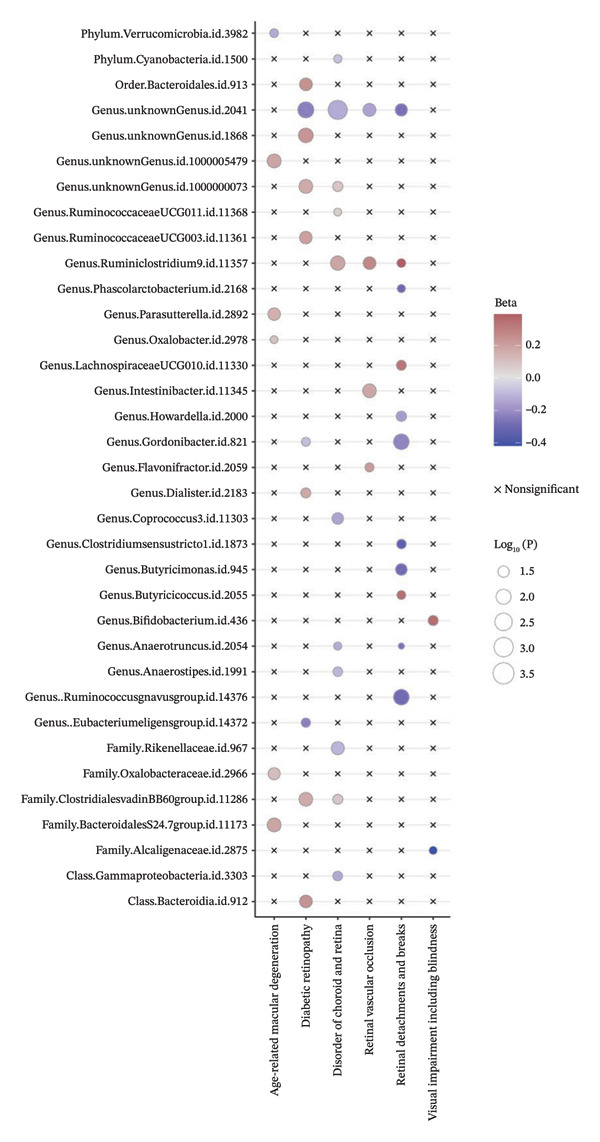
Balloon plot of the association between gut microbiota abundance and outcomes according to the IVW‐MR tests. Associations at *p* value > 0.05 are depicted with crosses.

**FIGURE 4:**
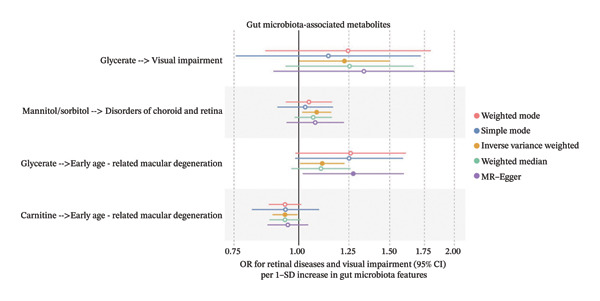
Forest plot of the associations between gut microbiota–associated metabolites and outcomes which were consistent in direction from MR analyses.

We performed two‐sample MR analysis on gut microbiota as exposures with relevant outcomes of retinal diseases and visual impairment by screening IVs of 211 bacteria separately. Of 1266 exposure–outcome IVW‐MR tests, 59 passed a significance threshold of 0.05 (*p* < 0.05), while 15 of those were excluded for the opposite effects among five MR analyses. A total of 44 exposure–outcome IVW‐MR tests were finally included in the analysis (Figure 3), and 35 (2 phyla, 2 classes, 1 order, 5 families, and 25 genera) remained after removing duplicates. In addition, among 28 selected gut microbiota–associated metabolites as exposures, 5 passed a significance threshold of 0.05, and 4 were consistent in direction from five MR estimates (Figure 4). All the primary two‐sample MR results are listed in Supporting Table S3. Results for outcomes with a limited number of cases should be interpreted with caution, as the lower statistical power may reduce the ability to detect small causal effects.

### 3.1. Effect of Gut Microbial Abundance on Retinal Diseases and Visual Impairment via Two‐Sample MR Analysis (Locus‐Wide Significance, *p* < 1 × 10^−5^)

#### 3.1.1. DR

This study identified eleven causal relationships between gut microbiota and the risk of DR (Figure 3). Higher genetically predicted abundances of Order *Bacteroidales*, Class *Bacteroidia*, Family *ClostridialesvadinBB60group*, Genus *Dialister*, Genus *RuminococcaceaeUCG003*, Genus *id.1000000073*, and Genus *id.1868* were associated with a higher risk of DR. In contrast, Genus *Eubacteriumeligensgroup*, Genus *Gordonibacter*, Genus *LachnospiraceaeNK4A136group*, and Genus *id.2041* were associated with a lower risk. The MR‒Egger intercept and MR‐PRESSO tests showed that there was no horizontal pleiotropy or outliers (*p* > 0.05); furthermore, the results of Cochrane’s *Q* test showed that no obvious heterogeneity was found in the selected SNPs (*p* > 0.05) (Supporting Table S4). Nonetheless, the leave‐one‐out method (Supporting Figure S1) demonstrated that some single SNPs might dominate the positive results for the above‐exposed microbiota, including Order *Bacteroidales*, Class *Bacteroidia*, Genus *Dialister*, Genus *Eubacteriumeligensgroup*, Genus *Gordonibacter*, and Genus *LachnospiraceaeNK4A136group*.

#### 3.1.2. eAMD

A causal association with eAMD was found for only six microbiotas. Higher genetically predicted abundances of Family *BacteroidalesS24.7group*, Genus *Oxalobacter*, Family *Oxalobacteraceae*, Genus *Parasutterella*, and Genus *id.1000005479* were associated with an increase in the incidence of eAMD (Figure 3), while Phylum *Verrucomicrobia* was associated with a decrease in the incidence of eAMD. No horizontal pleiotropy or outliers were observed according to the results of the MR‒Egger intercept and MR‐PRESSO tests (*p* > 0.05), and the outcomes from Cochrane’s *Q* test revealed no significant heterogeneity (Supporting Table S4) (*p* > 0.05). Eventually, the leave‐one‐out method (Supporting Figure S2) indicated that only Family *Bacteroidales S24.7 group* and Genus *id. 1000005479* achieved stable results after excluding the SNP on an individual basis.

#### 3.1.3. RDs/RBs

Three genetically predicted gut microbiotas were associated with an increased risk of RDs/RBs (Figure 3), including the genera *Flavonifractor*, *Intestinibacter*, and *Ruminiclostridium9*, whereas only Genus *id.2041* was associated with a reduced risk of RDs/RBs. The MR‒Egger intercept and MR‐PRESSO tests showed that there was no horizontal pleiotropy (*p* > 0.05), and again, no obvious heterogeneity was found according to Cochrane’s *Q* test (Supporting Table S4) (*p* > 0.05). The leave‐one‐out method showed that except for Genus *Intestinibacter* and Genus *id.2041*, there may be some bias in other genetic predictions (Supporting Figure S3).

#### 3.1.4. RVO

Eleven causal associations between gut microbiota and RVO were found (Figure 3). Higher genetically predicted abundances of Genus *Anaerotruncus*, Genus *Butyricicoccus*, Genus *Butyricimonas*, Genus *Clostridiumsensustricto1*, Genus *Ruminococcusgnavusgroup*, Genus *Gordonibacter*, Genus *Howardella,* Genus *LachnospiraceaeUCG010*, Genus *Phascolarctobacterium*, Genus *Ruminiclostridium9*, and Genus *id.2041* were associated with a decrease in the risk of RVO. Nevertheless, the genera *Butyricicoccus*, *LachnospiraceaeUCG010*, and *Ruminiclostridium9* were associated with an increase in the risk of RVO. The MR‒Egger intercept and MR‐PRESSO tests indicated that there was no horizontal pleiotropy or outliers (*p* > 0.05); additionally, no obvious heterogeneity was found according to the results of Cochrane’s *Q* test (Supporting Table S4) (*p* > 0.05). In addition, the leave‐one‐out method (Supporting Figure S4) hinted that in addition to Genus *Ruminococcusgnavusgroup*, Genus *Gordonibacter*, and Genus *id.2041*, some single SNPs were probably unable to achieve stable results.

#### 3.1.5. D‐C/R

Eleven causal relationships were found according to IVW analysis (Figure 3). Higher genetically predicted abundances of Family *ClostridialesvadinBB60group*, Genus *Ruminiclostridium9*, Genus *RuminococcaceaeUCG011*, and Genus *id.1000000073* were associated with an increase in the incidence of D‐C/R, whereas Genus *Anaerostipes*, Genus *Anaerotruncus*, Genus *Coprococcus3*, Phylum *Cyanobacteria*, Class *Gammaproteobacteria*, Family *Rikenellaceae*, and Genus *id.2041* were associated with a decrease in the incidence of D‐C/R. MR‒Egger intercept and MR‐PRESSO tests showed that there was no horizontal pleiotropy or outliers (*p* > 0.05); apart from Genus *Anaerotruncus*, no obvious heterogeneity was found according to results of Cochrane’s *Q* test (Supporting Table S4) (*p* > 0.05). In addition, the leave‐one‐out method (Supporting Figure S5) showed that except for Family *Rikenellaceae*, Genus *Ruminiclostridium9*, and Genus *id.2041*, other single SNPs might dominate the positive results.

#### 3.1.6. Visual Impairment

A higher genetically predicted abundance of (Figure 3) Genus *Bifidobacterium* was associated with an increased risk of visual impairment; in contrast, Family *Alcaligenaceae* was associated with a reduced risk of visual impairment. No significant heterogeneity or horizontal pleiotropy was found according to Cochrane’s *Q*, MR‒Egger intercept, and MR‐PRESSO tests (Supporting Table S4). After removing each SNP separately, none of the results remained stable (Supporting Figure S6).

### 3.2. Effect of Gut‐Associated Metabolites on Retinal Diseases and Visual Impairment via Two‐Simple MR Analysis (Locus‐Wide Significance, *p* < 5 × 10^−6^)

Among the 28 selected metabolites associated with gut microbiota and their functional pathways, a causal relationship was determined for four metabolites to influence some retinal diseases. IVW‐MR analyses were performed for each of the outcomes under study, and the positive results are displayed in forest plots (Figure 4). Dichotomous traits are reported on an OR scale, and continuous traits are reported on a 1‐SD scale. Dots depict the point estimate, and horizontal bars depict the 95% confidence interval (CI). Three metabolites—carnitine, glycerate, and mannitol/sorbitol—were identified as associated with outcomes. Carnitine and glycerate levels showed an association with eAMD, whereas mannitol/sorbitol levels showed a causal association with D‐C/R. In addition, glycerate levels were associated with an increased risk of visual impairment. Notably, no explicit association of the reverse relationship was found. Altogether, some gut‐derived metabolites were identified that may be causally associated with retinal diseases and visual impairment. However, the possibility of spurious associations cannot be completely excluded, as none of the metabolites showed association with the outcomes of interest after correction for multiple factors.

**FIGURE 5:**
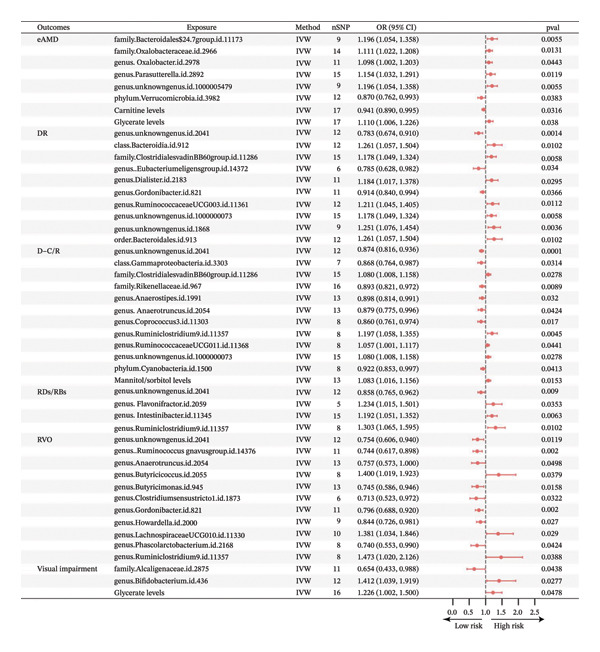
Forest plot of causal effects of gut microbiota and associated metabolites on retinal diseases and visual impairment.

A supplementary table summarizing the associations and effect sizes between all primary exposures and outcomes in this study is presented in Figure 5.

### 3.3. Bonferroni‐Corrected Test and Reverse Analysis

The results from the Bonferroni‐corrected test revealed that a higher abundance of Genus *id.2041* retained a strong causal relationship with D‐C/R (OR = 0.874, 95% CI: 0.816–0.936, *p* = 1.10e − 04, *q* = 2.42e − 02). Reverse analysis (Supporting Table S6) demonstrated that D‐C/R may lead to a higher abundance of Family *Rikenellaceae* (*p* = 0.027), but we did not observe an explicit association for any other gut microbiota or associated metabolites (*p* > 0.05). Altogether, this analysis identified some gut microbiota that may be associated with retinal diseases and visual impairment.

### 3.4. Multivariable MR Analysis

**FIGURE 6:**
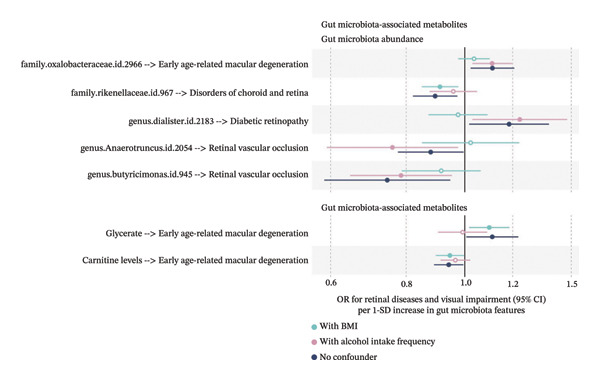
Multivariable MR results for BMI and alcohol intake frequency as confounding factors.

Obesity and alcohol intake frequency have been recently regarded as major confounding factors in microbiome–disease associations [39]. Multivariable MR analysis was performed on the 48 promising associations to determine whether the causal effects were robust to the inclusion of obesity and alcohol intake frequency (Supporting Table S7). This analysis suggests that the seven reported associations may be partially influenced by factors, such as body mass index (BMI) or alcohol consumption (Figure 6). Dichotomous traits are reported on an OR scale, and continuous traits are reported on a 1‐SD scale. Dots depict the point estimate, and horizontal bars depict the 95% CI. For instance, the causal effect of Family *Oxalobacteraceae* abundance on eAMD remained valid (OR = 1.11, 95% CI 1.02–1.21, *p* = 1.3e − 02) despite the inclusion of alcohol intake frequency as a covariate, whereas the aforementioned association did not exist when only the covariate BMI was included. However, the *F*‐values of the above results are all less than 10, indicating a weak explanatory power of the IVs. Apart from the seven associations, upon the inclusion of BMI or alcohol intake frequency as covariates separately, the causal effect of exposures on outcomes attenuated toward null.

## 4. Discussion

Leveraging new and large GWAS data, our study is a comprehensive MR study that explores the causal relationship between intestinal microorganism features and the occurrence of major retinal diseases as well as visual impairment, based on gene prediction. Contents covered in our study included the major retinal diseases, ranging from age‐related to metabolism‐related, from idiopathic to secondary, and from anatomical to functional. Previous studies that investigated the association between gut microbiota and retinal diseases were observational studies or randomized controlled trials, including clinical research and animal models. The microbiome as an exposure phenotype limitedly explained by genotype is strict enough due to the robust calculation of MR statistical power. Since alleles were randomly assigned at conception, our MR analysis minimizes biases from confounding and reverse causality [40]. Although our sample may restrict the generalizability of the results to specific ethnic groups, it was restricted to individuals of European ancestry in an effort to reduce population stratification bias. Furthermore, for human observational studies, multiple confounding factors might create spurious association between gut microbiota features and retinal diseases, including sex, age, geography, diet, obesity, and alcohol intake [41, 42]. In order to eliminate the interferential impact of lifestyle, our multivariable MR analysis adequately revealed the major covariates’ effects—BMI and alcohol intake frequency. As a result, the findings are more robust and offer reliable causal insights, which could inform future early‐stage interventions of retinal diseases by targeting specific intestinal microorganisms.

### 4.1. Potential Functions of Gut‐Derived Metabolites

Encouragingly, the change in gut microbiota‐associated metabolites and their effect on retinal health substantiated nutritional prophylaxis against retinal diseases. According to the assessment of targeted metabolomic profiles in normal whole mouse retina tissue [43], 114 of 171 metabolites were discovered to be involved in glucose metabolism, the tricarboxylic acid cycle, amino acids, nucleotides and their metabolites, tryptophan cycle metabolites, vitamins, and sugars and lipids/fatty acids, which is important for the design of future intervention studies on retina tissue. In DR metabolomics studies, purine metabolism, pyrimidine metabolism, arginine and proline metabolism, and glutamate metabolism are the most frequently reported differential pathways [44], and it has been reported that proliferative DR is associated with reduced diversity and altered composition of the gut microbiome and specific microbe–metabolite interplay [45]. Another nested, population‐based, case‒control study showed that DR samples had decreased levels of 1,5‐anhydroglucitol and increased levels of 1,5‐ gluconolactone, 2‐deoxyribonic acid, 3,4‐dihydroxybutyric acid, erythritol, gluconic acid, lactose/cellobiose, maltose/trehalose, mannose, ribose, and urea [46]. In AMD metabolomics studies, Rowan et al. revealed gut microbiota–associated cometabolites in a wild‐type mouse model, particularly serotonin, as protective against AMD features, and network analysis revealed a nexus of metabolites and microbiota that appear to act within a “gut–retina axis” to protect against diet‐ and age‐induced AMD features [47]. Metabolic pathway inferences suggested that the gut bacteria of AMD patients have reduced fatty acid elongation and increased L‐alanine fermentation, glutamate degradation, and arginine biosynthesis, which may plausibly affect retinal health. Changes in cellular metabolism underlie vitreoretinal pathologies, and the vitreous humor is considered a surrogate for retinal metabolomic analysis. In our MR analysis, carnitine levels presented a significant protective effect against eAMD, whereas significant alteration of the carnitine shuttle pathway has been confirmed in intermediate and neovascular AMD [48]. For RVO, no studies have investigated the influence of the gut microbiome on retinal vein occlusion to date. The class *Actinobacteria* and the species *Bifidobacterium adolescentis*, *Bifidobacterium bifidum*, *Bacteroides stercoris*, and *Faecalibacterium prausnitzii* were enriched in retinal artery occlusion patients [49], as well as the mevalonate pathway and methylerythritol phosphate pathway involved in cholesterol metabolism [50]. Evidence for an influence of the gut microbiome on retinal health is likely accumulating under cross‐interaction, and dysbiosis may lead to the development of D‐CR and visual impairment. For instance, glycerate levels pose a risk factor for eAMD and likewise have similar effects on visual impairment. Higher mannitol/sorbitol is one of the risk factors for D‐CR, hinting at the potential mechanism of multiple actions of metabolic processes. With a deeper understanding of the implications of gut‐derived metabolites on retinal health, targeted therapeutic approaches could be developed to achieve personalized options.

### 4.2. Presumably Outstanding Performance of Unknown Gut Microbiota

Among the notable findings in our MR study is the robust association between Genus id.2041 and a reduced risk of D‐C/R (OR = 0.874, *p* = 1.10e − 04), which persisted after Bonferroni’s correction. However, the precise taxonomic identity and functional characterization of Genus id.2041 remain elusive, as it represents an unclassified or poorly annotated microbial group within current reference databases. This ambiguity severely limits our ability to propose mechanistic explanations for its protective role. It is plausible that id.2041 may belong to an undersampled or fastidious bacterial lineage with anti‐inflammatory, metabolic, or barrier‐strengthening properties that indirectly influence retinal health via the gut–retina axis. Yet, without genomic, transcriptomic, or culture‐based validation, any functional inference remains speculative. Furthermore, the unknown nature of this genus poses a significant challenge to its potential translation into clinical biomarkers or therapeutic targets. Data mining for unknown gut microbiota has enlightening the significance for retinal disease therapeutics. Metagenomic dataset analysis can provide both taxonomic and functional information from complex microbial communities, guiding phenotypic studies aimed at understanding their potential roles in human health and disease [51]. Future studies should prioritize the isolation, sequencing, and phenotypic profiling of id.2041‐like organisms through culturomics, metagenomic binning, or single‐cell genomics to clarify its taxonomic placement and elucidate its role in retinal pathophysiology. Almeida et al. reported that 1952 uncultured bacterial species were discovered by reconstructing metagenome‐assembled genomes [52], substantially expanding the known species repertoire of the collective human gut microbiota, with a 281% increase in phylogenetic diversity. However, various strategies used for the analysis of metagenomic datasets rely on high‐quality reference databases, which highlights the need for extensive and well‐characterized collections of reference genomes [53]. Despite a new wave of culturing efforts (for example, culture omics has been developed to culture and identify unknown bacteria [54]), there is still a substantial but undetermined degree of unclassified microbial diversity within the gut ecosystem [52, 55]. Whereas unknown gut microbiota members may have eluded current isolating and culturing strategies for a variety of reasons (for instance, owing to lack of nutrients in growth media and being incapable of simulating veritable intestinal conditions), they are likely to perform crucial biological roles that remain undiscovered. A comprehensive catalog of representative genomes and isolates from the gut microbiome is essential to gain new mechanistic insights. In all, the effect of unknown gut microbiota on retinal diseases remains to be explored thoroughly.

### 4.3. The Interferential Impact of Lifestyle

Beyond specific gut microbiota and microbial metabolites, our findings also point to the potential influence of host lifestyle factors on the gut–retina axis. Our multivariable MR analyses revealed that the causal estimates for several nominal associations attenuated upon adjustment for BMI or alcohol intake frequency. This observation underscores the critical, potentially confounding role of lifestyle and metabolic factors in shaping the gut microbiome and its systemic effects. Epidemiological and intervention studies have consistently demonstrated that both obesity (high BMI) and alcohol consumption indirectly induce alterations in gut microbiota composition [56], often characterized by reduced overall diversity and enrichment of pro‐inflammatory or opportunistic taxa. These microbial shifts are frequently accompanied by increased intestinal permeability and systemic inflammation—key pathophysiological processes implicated in the development and progression of numerous retinal diseases. Therefore, the attenuation of MR signals after adjusting for these factors suggests that the observed statistical associations between certain gut microbes and retinal outcomes may not represent direct causal pathways. However, relying solely on less quantity of SNPs and introducing low F‐values results in a certain degree of bias in the analysis. At this point, we need to be very cautious in interpreting the results. Still, our findings indicate the necessity of accounting for major lifestyle confounders in microbiome–disease association studies and suggest that interventions targeting obesity or excessive alcohol use could beneficially modulate the gut microbiome, thereby potentially exerting a protective effect on retinal health through the gut–retina axis. Future research should integrate detailed lifestyle data with multiomics approaches to dissect the complex interplay between host behavior, gut microbiota, and ocular pathology. In addition, biomarkers, such as blood glucose and blood lipid level, are critical for understanding the onset and progression of retinal disease, which be considered as key variables in the previous MR studies. However, our MR study focused on lifestyle habits and nutritional status instead of emphasizing the confounding of biological indicators. In future research, comprehensive blood traits should be taken into account to enhance the accuracy of causal inferences.

### 4.4. Comparisons With Other Studies

Diet regulates the immune system by altering gut microbiota and their metabolites, affecting the progression of retinal diseases. First, there is a possible increase in the *Bacteroidetes* to *Firmicutes* ratio in elderly individuals [57], and the ratio has been shown to have an effect on several diseases that also affect AMD [58]. Our MR study revealed a risk for AMD with high *Bacteroidetes* abundance (Family *BacteroidalesS24.7group*) at the genomic level, whereas it did not show a risk for AMD with high abundances of *Oscillibacter* [59], *Anaerotruncus* [60, 61], *Firmicutes*, or *Proteobacteria* [62]. Additionally, significant increases in the proportions of *Acidaminococcus*, *Escherichia*, and *Enterobacter* appear among the microbiota of patients with DR [63]. Associated with a high‐fat diet and inflammation, the ratio of *Bacteroidetes* to *Firmicutes*, as a genetic background that negatively affects aged retinas, was found to be significantly different between DR patients and controls [64]. In addition, several species, including *Actinobacteria*, *Bifidobacterium spp.*, *Bacteroides stercoris*, and *Faecalibacterium prausnitzii,* are relatively enriched in patients with retinal artery occlusion, whereas others, such as *Odoribacter*, *Parasutterella*, or *Lachnospiraceae*, are significantly depleted [49]. The association between relative gut microbiota abundance and trimethylamine‐N‐oxide levels is also associated with an increased risk of retinal diseases [65], especially retinal vessel injury. It has been reported that gut microbiota and associated metabolite trimethylamine oxide are involved in the process of RVO [66]. For D‐C/R, the results from Cochrane’s *Q* test indicated that Genus *Anaerotruncus* might have obvious heterogeneity (*p* < 0.05); however, with the IVW‐MR tests, it was proven that the relationship between Genus *Anaerotruncus* and D‐C/R was dependable to some extent. The complicated and inconsistent results for the Genus *Anaerotruncus* may hint that its abundance is a consequence of disease states instead of merely a causal factor. Altogether, the results of this study suggest that previously reported associations between human gut microbiota and human diseases might have been due to biases, such as reverse causality or confounding, and that the impact of gut microbiota on D‐C/R may not be as absolute as considered. Gut microbiota alterations could be consequences of disease states rather than causal factors. Since visual impairment is usually regarded as the ultimate outcome of many eye diseases, the causal relationship between gut microbiota features and visual impairment may overlook the indirect effects of these features on primary retinal diseases. Nevertheless, the findings of this study provide insights and perspectives for further deliberation of relevant metabolic effects on visual impairment. Altogether, gut microbiota and their metabolites may be new targets for the treatment of eye diseases.

Nevertheless, the causal relationships between gut microbiota and retinal diseases reported in previous MR studies are partially incompatible with our results [34], since data sources have a strong influence on the selection bias. Confounding bias exists because the crosstalk between the gut microbiome and the retina is often coordinated by multiple factors, and the role of a single gut microbiota in causing disease may not be as prominent as previously thought. The large proportion of negative findings after the Bonferroni‐corrected test is in line with recent literature, showing a positive publication bias in the microbiome literature [67], which occurs when studies are not adequately corrected for multiple factors, which can be identified through attempts to and subsequent failure to replicate the findings [68]. All of the abovementioned factors highlight the importance of using multiple methods to address causal research questions.

### 4.5. Strengths and Limitations

A key strength of our MR study lies in using the largest publicly available GWAS datasets. Another advantage is that the sample primarily consisted of individuals of European ancestry, which helps minimize bias from population stratification, though this limits the generalizability of the results to other ethnic groups. Furthermore, both genetic and environmental factors influence retinal disease symptoms. While genotype and environmental factors have independent effects, the impact of environmental factors can vary based on genetic makeup. To account for these complexities, we conducted an MR study to control for confounders commonly found in epidemiological research.

It is of great importance to acknowledge the limitations of our study. Identifying reliable genetic instruments for microbial species remains a challenge. First, the high variability in microbiome composition between individuals reduces the statistical power of microbiome GWAS analyses. The vast majority of GWAS dataset was derived from individuals of European ancestry as shown in Table S1; accordingly, the findings may not be directly generalizable to other ancestral populations. Second, because the phenotype is influenced by multiple genes, it becomes a complex trait with many small‐effect variants, which may be more susceptible to pleiotropy. Third, since MR analysis is based on untestable assumptions, further experimental and clinical validation studies are crucial to test the clinical significance of microbial species. For outcomes with lower power, nonsignificant findings should be interpreted cautiously, as true causal effects may have been undetected due to limited sample size.

## 5. Conclusions

Through MR analysis of the causal relationship between gut microbiota features and six phenotypes, we identified 47 nominal causalities and 1 strong causal association. Among them, Genus *id.2041* was strongly associated with D‐C/R. Our research identified specific microbiota using genetic prediction that warrant further investigation for future clinical application. As the field of microbiome research matures, the use of a larger microbiome GWAS dataset taking advantage of the discriminatory potential of the full 16S RNA gene is warranted to fully elucidate the role of gut microbiota in the etiology of retinal diseases.

**Nomenclature**
GWAS: Genome‐wide association study
SNP: Single‐nucleotide polymorphism
MR: Mendelian randomization
IVW: Inverse variance weighted
DR: Diabetic retinopathy
eAMD: Early age‐related macular degeneration
RDs/RBs: Retinal detachments and breaks
RVO: Retinal vascular occlusion
D‐C/R: Disorders of the choroid and retina
IAMDGC: International AMD Genomics Consortium
UKBB: UK Biobank

## Author Contributions

Chuyao Yu designed the structure of the manuscript and wrote the manuscript. Li Dong, Ruiheng Zhang, Heyan Li, Xuhan Shi, Haotian Wu, Wenda Zhou, and Yitong Li help collect the information. Li Dong and Wen‐Bin Wei revised the manuscript. All the authors participated in planning, execution, and analysis.

## Funding

The study was supported by the National Natural Science Foundation of China (82220108017, 82141128, 82401283); the Capital Health Research and Development of Special (2024‐1‐2052, 2026‐4‐20510); Science & Technology Project of Beijing Municipal Science & Technology Commission (Z201100005520045); Sanming Project of Medicine in Shenzhen (No. SZSM202311018); R&D Program of Beijing Municipal Education Commission (No. KM202410025011); 2025 Project of Beijing Education Science “14th Five‐Year Plan” (CDGB25537); and the priming scientific research foundation for the junior researcher in Beijing Tongren Hospital, Capital Medical University (No. 2023‐YJJ‐ZZL‐003).

## Disclosure

All authors have read and approved the final submitted version. This manuscript is an original submission. The current version has been revised, instead of a duplicate submission. This work has not been published elsewhere.

## Ethics Statement

Ethical review and approval were not required for the study on human participants in accordance with the local legislation and institutional requirements. Written informed consent for participation was not required for this study in accordance with the national legislation and the institutional requirements.

## Consent

Please see the Ethics Statement.

## Conflicts of Interest

The authors declare no conflicts of interest.

## Supporting Information

Additional supporting information can be found online in the Supporting Information section.

## Supporting information


**Supporting Information 1** Supporting File 1: Supporting Tables. Table S1: Data source of MR analysis; Table S2: The selection of gut microbiota associated metabolites as exposures; Table S3: Primary MR results and power; Table S4: Sensitivity analysis for MR results; Table S5: Harmonized dataset for measurable exposure–outcome and each F‐statistics; Table S6: Reverse MR results; Table S7: Multivariable MR results.

**Supporting Information 2** Supporting File 2: Supporting Figures. Supporting Figure S1–S6: Leave‐one‐out plots of significant and nominal significant estimates from genetically predicted gut microbiota on DR, eAMD, RD/RB, RVO, D‐C/R and Visual impairment; Supporting Figure S7–S12: Scatter plots of significant and nominal significant estimates from genetically predicted gut microbiota on DR, eAMD, RD/RB, RVO, D‐C/R and Visual impairment; Supporting Figure S13–S18: Funnel plots of significant and nominal significant estimates from genetically predicted gut microbiota on DR, eAMD, RD/RB, RVO, D‐C/R and Visual impairment.

## Data Availability

The original contributions presented in the study are openly available in the FinnGen study (https://www.finngen.fi/en), MiBioGen consortium (https://mibiogen.gcc.rug.nl/), GWAS Catalog (https://www.ebi.ac.uk/gwas/studies/GCST010723; https://www.ebi.ac.uk/gwas/publications/36635386), and IEU OpenGWAS Project (https://gwas.mrcieu.ac.uk/datasets/ukb-b-5779/; https://gwas.mrcieu.ac.uk/datasets/ukb-b-2303/). Further inquiries can be directed to the corresponding author. The other datasets generated and/or analyzed during this study are publicly available and included in this published article and its supplementary information files.
