# Supplementary material for: Causal Effects of Gut Microbiota and Associated Metabolites on Retinal Diseases and Visual Impairment: A Mendelian Randomization Study
Source: J Ophthalmol. 2026 Jun 17;2026:4233490. doi: 10.1155/joph/4233490 (PMC13273394; doi:10.1155/joph/4233490)
Supplement: Supplementary file 2 — Supporting Information 2 Supporting File 2: Supporting Figures. Supporting Figure S1–S6: Leave‐one‐out plots of significant and nominal significant estimates from genetically predicted gut microbiota on DR, eAMD, RD/RB, RVO, D‐C/R and Visual impairment; Supporting Figure S7–S12: Scatter plots of significant and nominal significant estimates from genetically predicted gut microbiota on DR, eAMD, RD/RB, RVO, D‐C/R and Visual impairment; Supporting Figure S13–S18: Funnel plots of significant and nominal significant estimates from genetically predicted gut microbiota on DR, eAMD, RD/RB, RVO, D‐C/R and Visual impairment. [file JOPH-2026-4233490-s002.docx]

**Supplementary Figures**

**Causal Effects of Gut Microbiota and** **Associated Metabolites on Retinal Diseases and Visual Impairment: A Mendelian Randomization Study**

**Chuyao Yu^1^, Li Dong^1^, Ruiheng Zhang^1^, Heyan Li^1^, Xuhan Shi^1^, Haotian Wu^1^, Wenda Zhou^1^, Yitong Li^1^, Wen-Bin Wei^1^***

^1^ Beijing Tongren Eye Center, Beijing key Laboratory of Intraocular Tumor Diagnosis and Treatment, Beijing Ophthalmology＆Visual Sciences Key Lab, Medical Artificial Intelligence Research and Verification Key Laboratory of the Ministry of Industry and Information Technology, Beijing Tongren Hospital, Capital Medical University, Beijing 100730, China.

*Correspondence: Wen-Bin Wei: [weiwenbintr@163.com](mailto:weiwenbintr@163.com;Z)

**Supplementary** **Figure S1**

Leave-one-out plots of significant and nominal significant estimates from genetically predicted gut microbiota { (A) Class.Bacteroidia.id.912; (B) Family.ClostridialesvadinBB60group.id.11286; (C) Genus..Eubacteriumeligensgroup.id.14372; (D) Genus.Dialister.id.2183; (E) Genus.Gordonibacter.id.821; (F) Genus.RuminococcaceaeUCG003.id.11361; (G) Genus.unknowngenus.id.1868; (H) Genus.unknowngenus.id.2041; (I) Genus.unknowngenus.id.1000000073; (J) Order.Bacteroidales.id.913 } on DR.


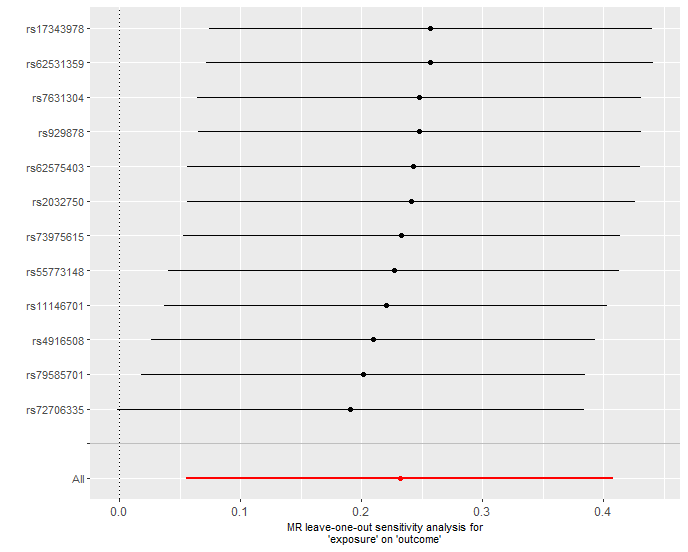

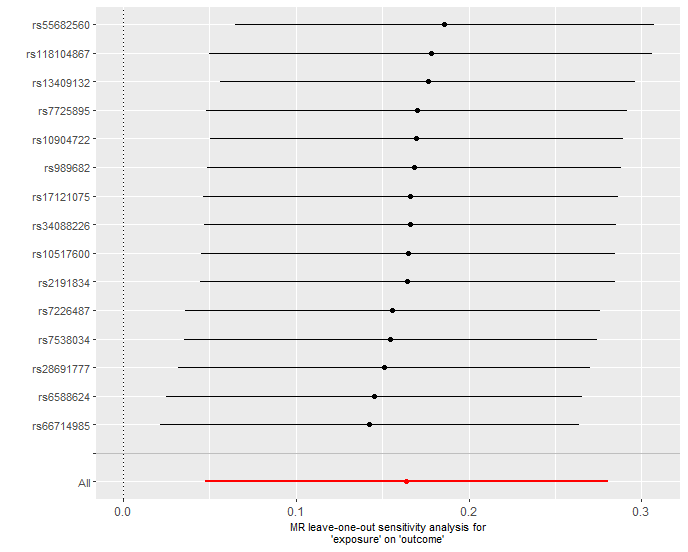

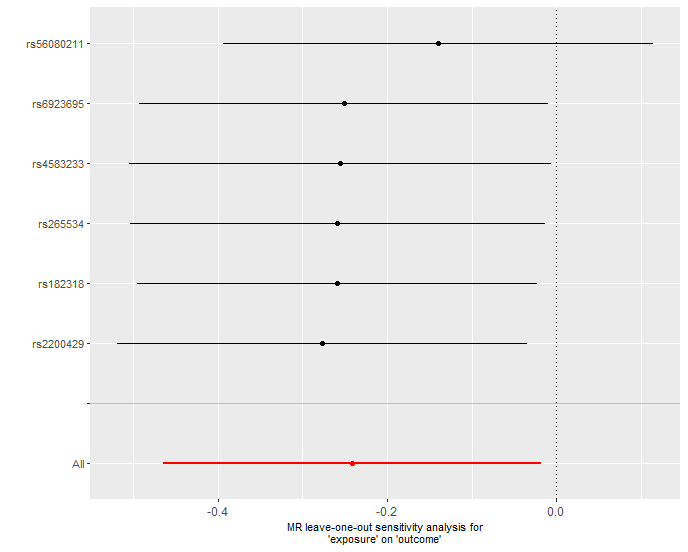

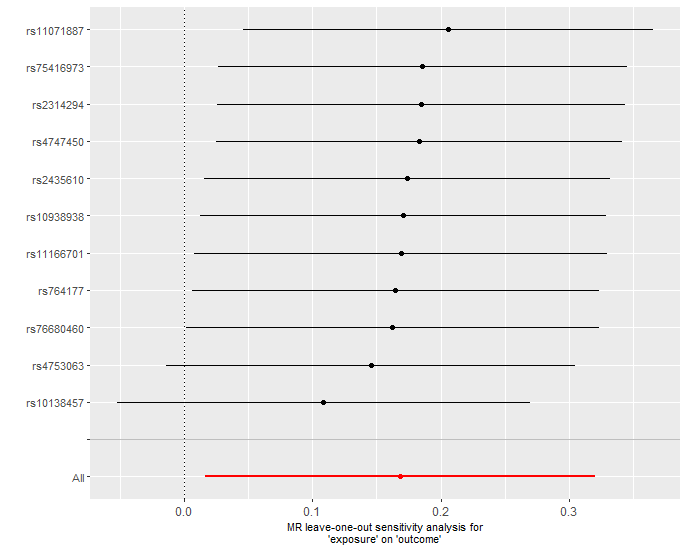

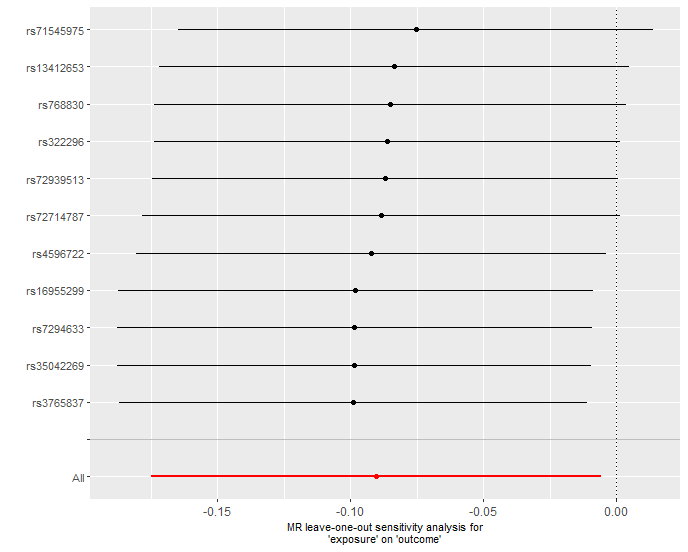

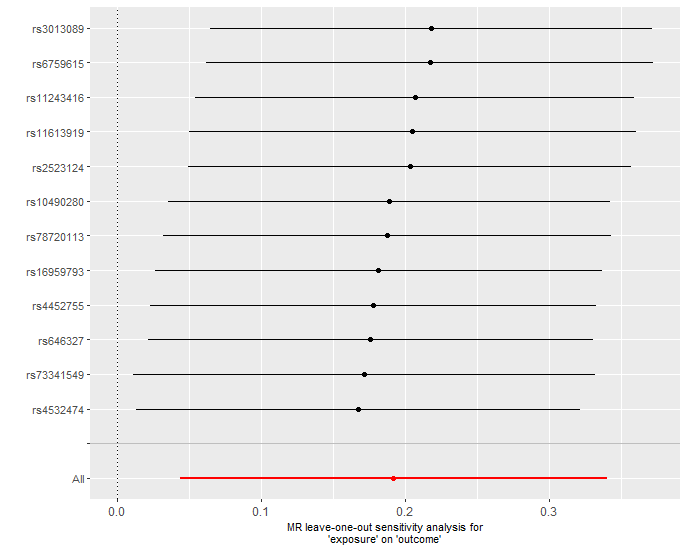

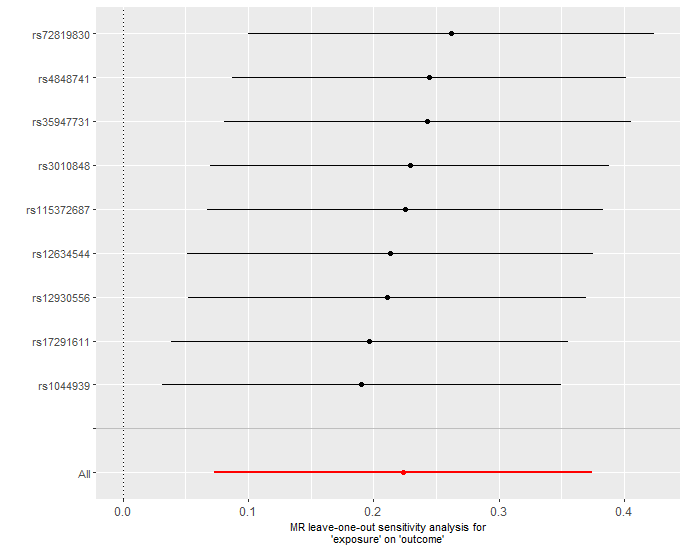

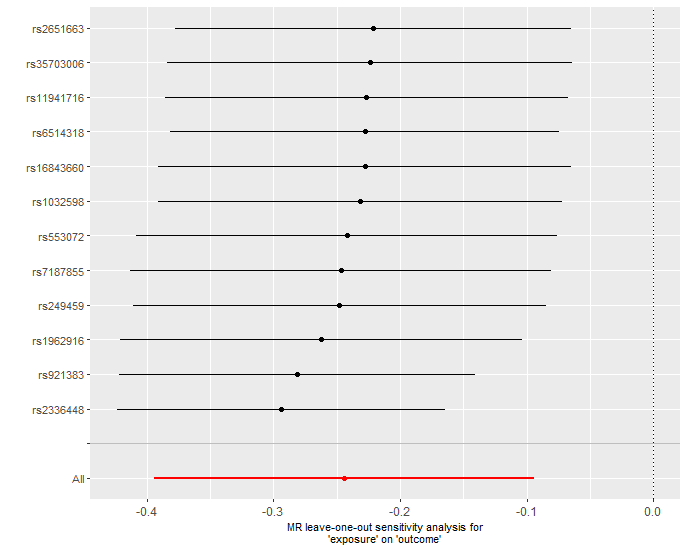

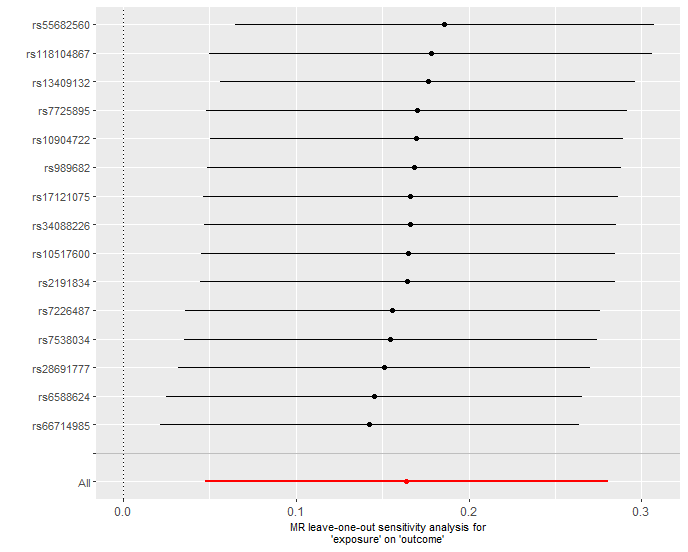

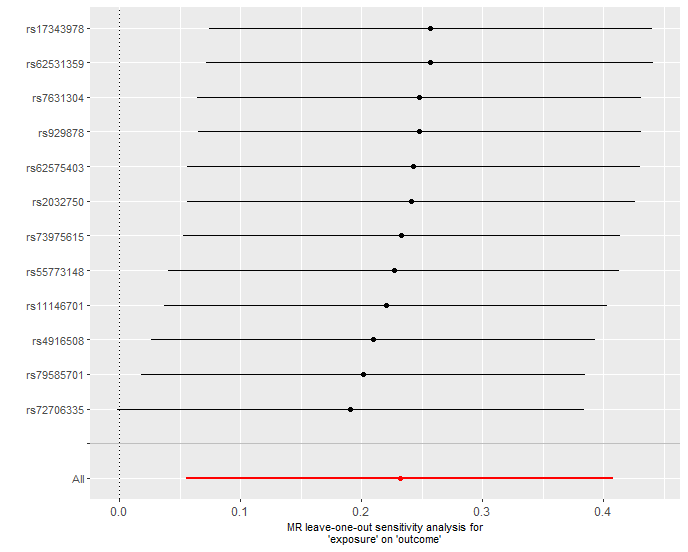


**A**

**B**

**C**

**D**

**E**

**F**

**G**

**H**

**I**

**J**

**Supplementary Figure S2**

Leave-one-out plots of significant and nominal significant estimates from genetically predicted gut microbiota { (A) Family.BacteroidalesS24.7group.id.11173; (B) Family.Oxalobacteraceae.id.2966; (C) Genus.Oxalobacter.id.2978; (D) Genus.Parasutterella.id.2892; (E) Genus.unknowngenus.id.1000005479; (F) Phylum.Verrucomicrobia.id.3982 } on eAMD.


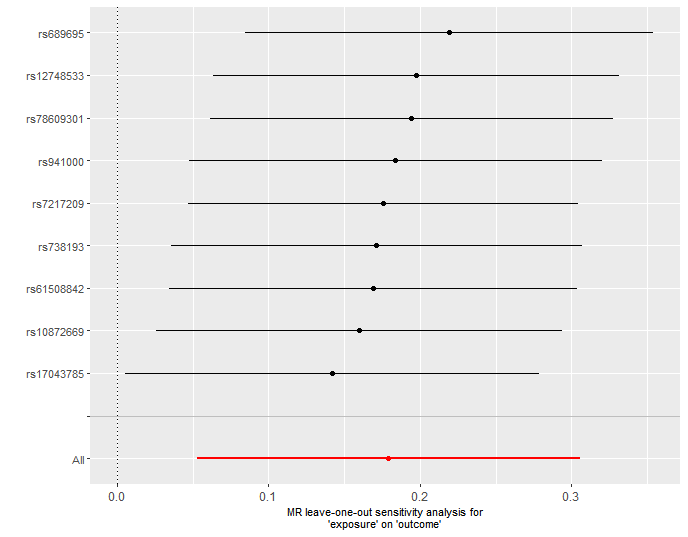

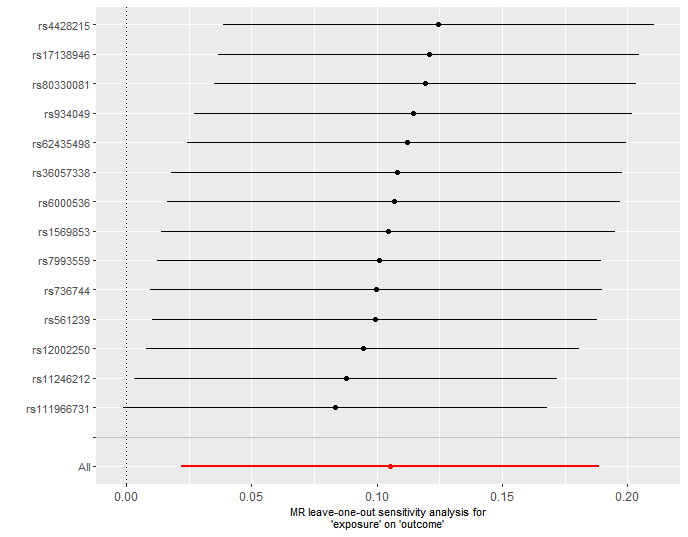

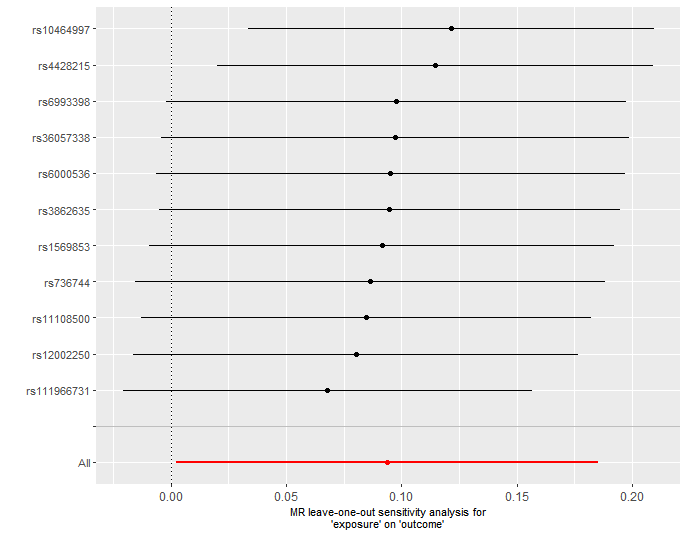

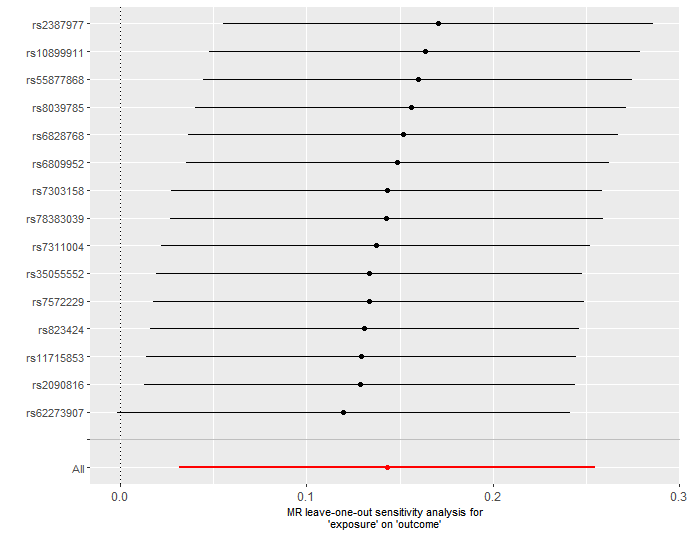

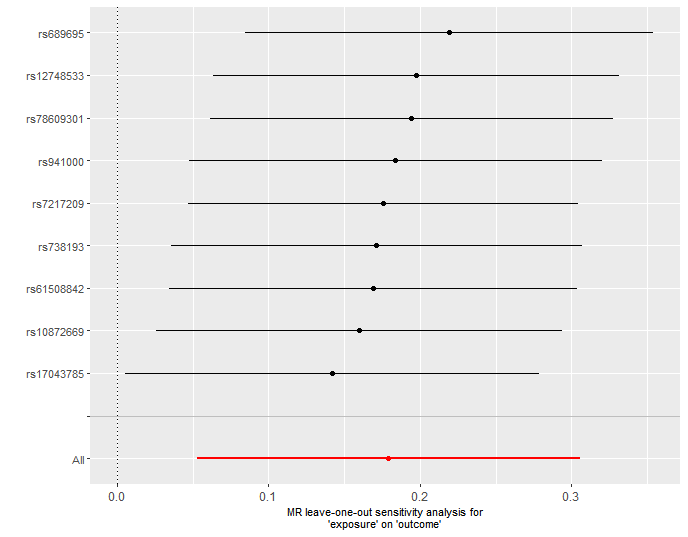

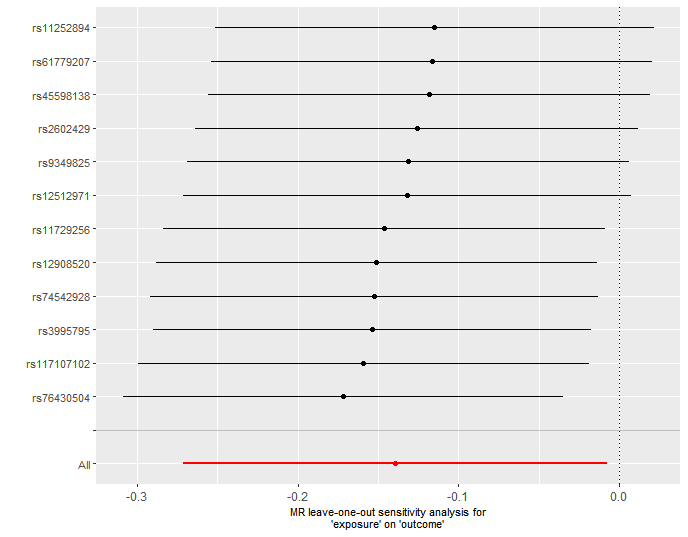


**A**

**B**

**C**

**D**

**E**

**F**

**Supplementary Figure S3**

Leave-one-out plots of significant and nominal significant estimates from genetically predicted gut microbiota { (A) Genus.Flavonifractor.id.2059; (B) Genus.Intestinibacter.id.11345; (C) Genus.Ruminiclostridium9.id.11357; (D) Genus.unknowngenus.id.2041 } on RD/RB.


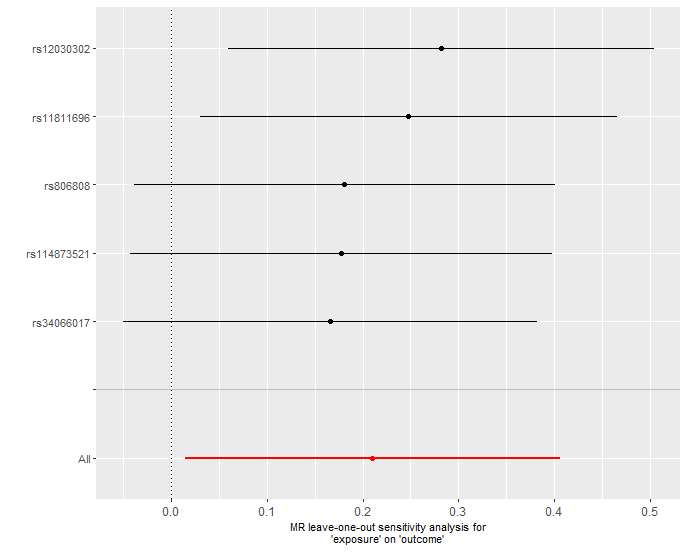

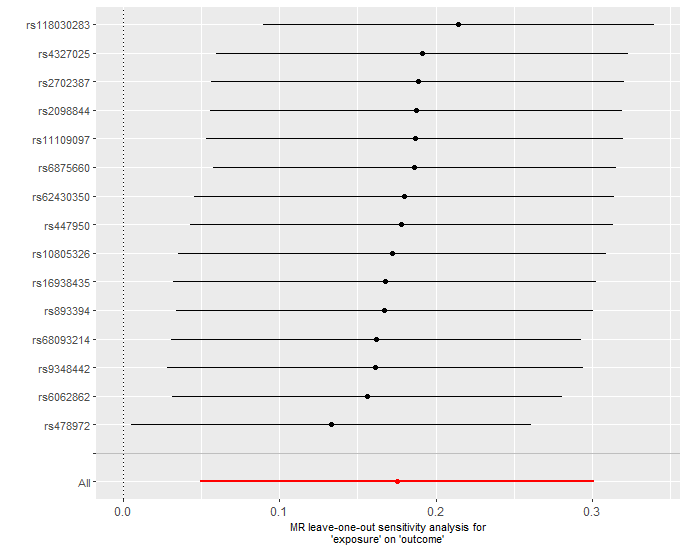

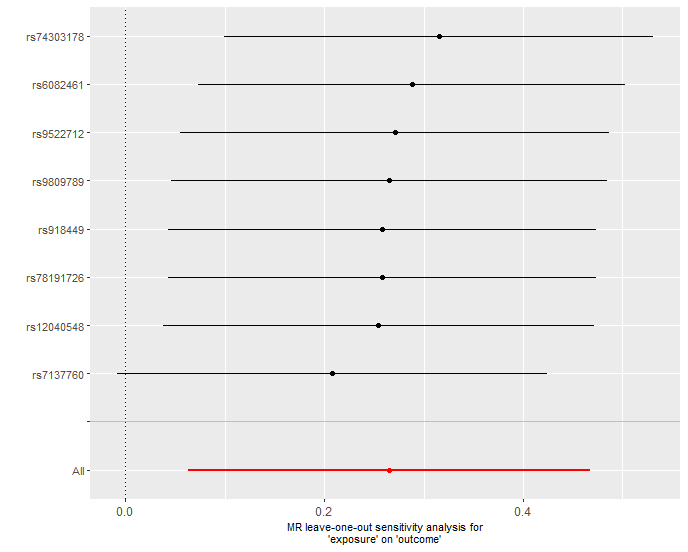

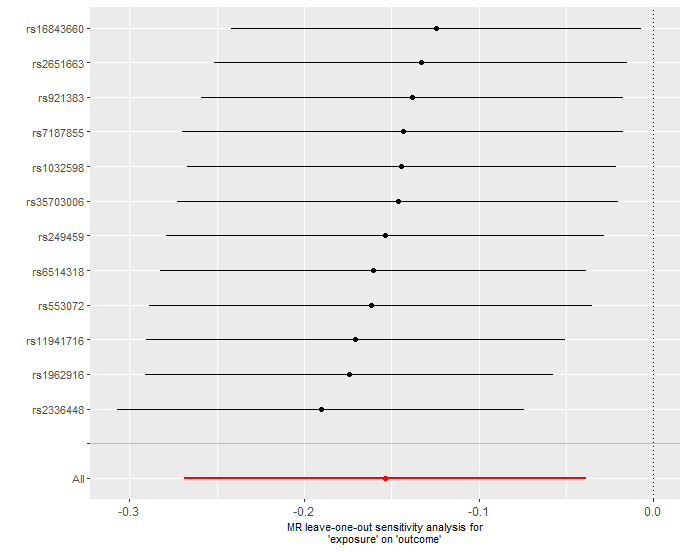


**A**

**B**

**C**

**D**

**Supplementary Figure S4**

Leave-one-out plots of significant and nominal significant estimates from genetically predicted gut microbiota { (A) Genus..Ruminococcusgnavusgroup.id.14376; (B) Genus.Anaerotruncus.id.2054; (C)Genus.Butyricicoccus.id.2055; (D) Genus.Butyricimonas.id.945; (E) Genus.Clostridiumsensustricto1.id.1873; (F) Genus.Gordonibacter.id.821; (G) Genus.Howardella.id.2000; (H) Genus.LachnospiraceaeUCG010.id.11330; (I) Genus.Phascolarctobacterium.id.2168; (J) Genus.Ruminiclostridium9.id.11357 ; (K) Genus.unknowngenus.id.2041 } on RVO.


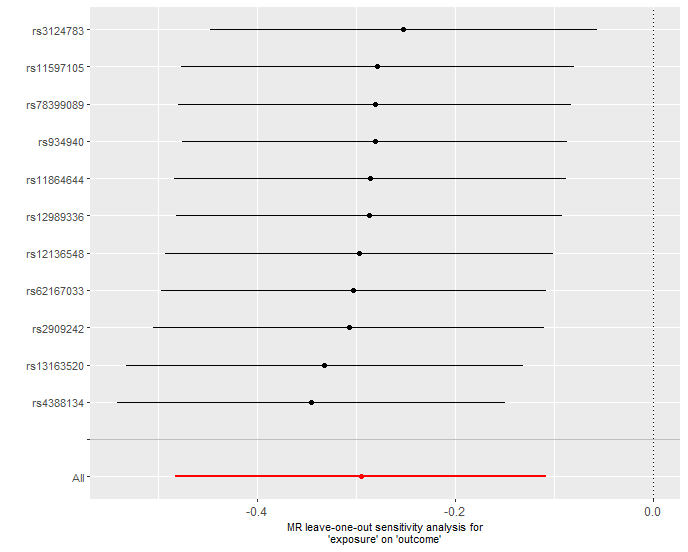

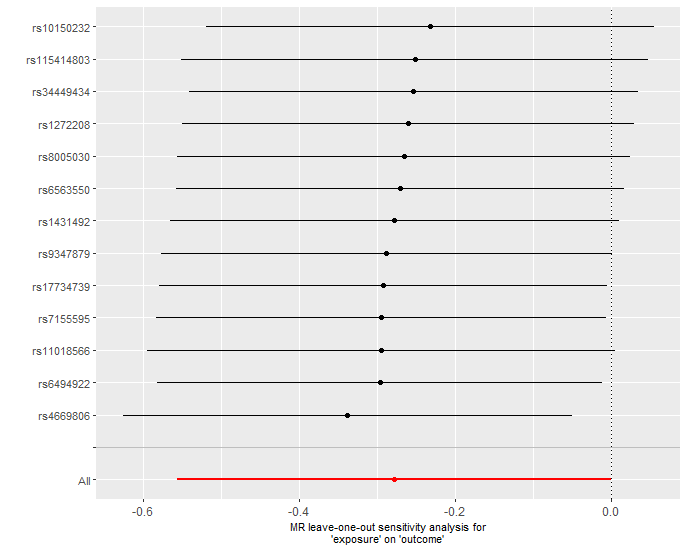

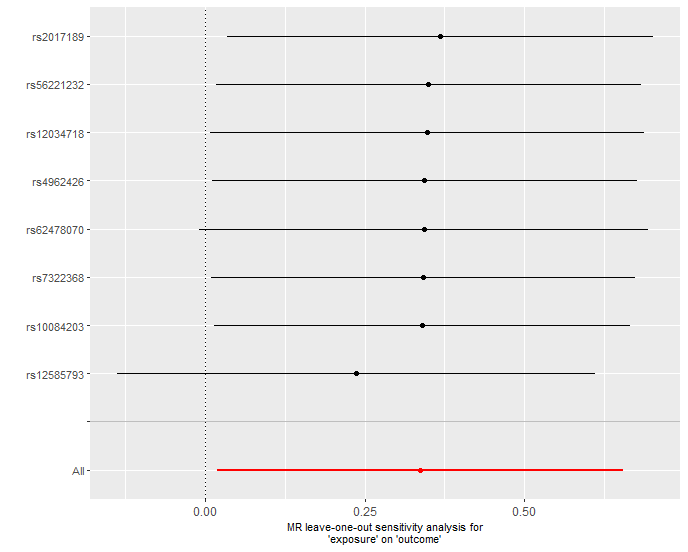

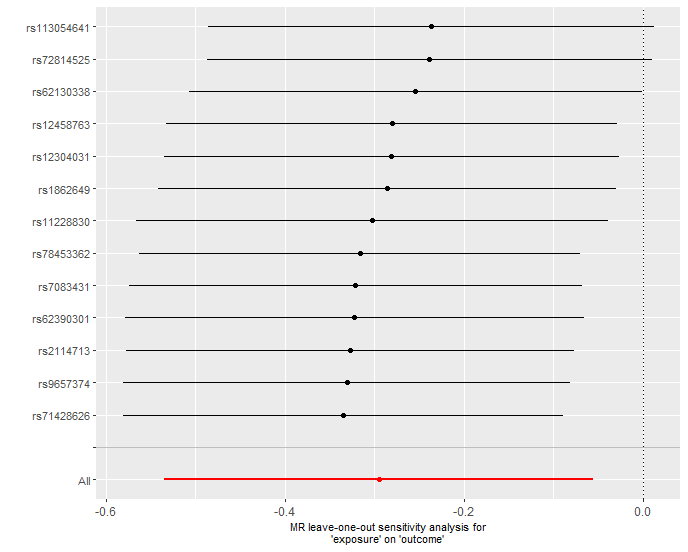

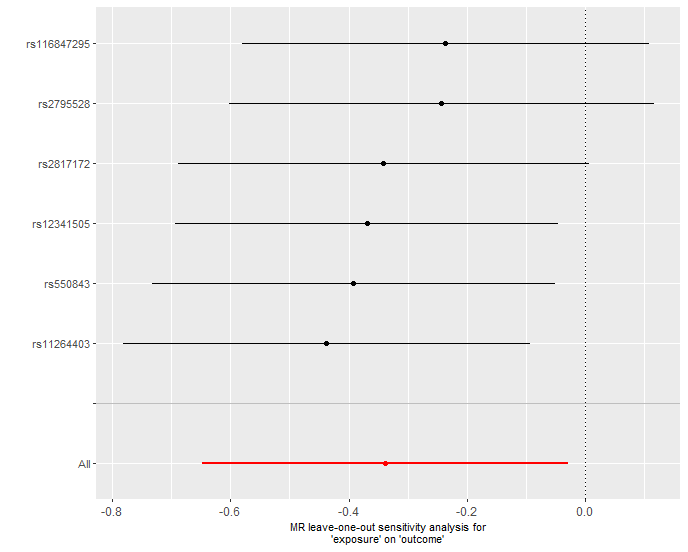

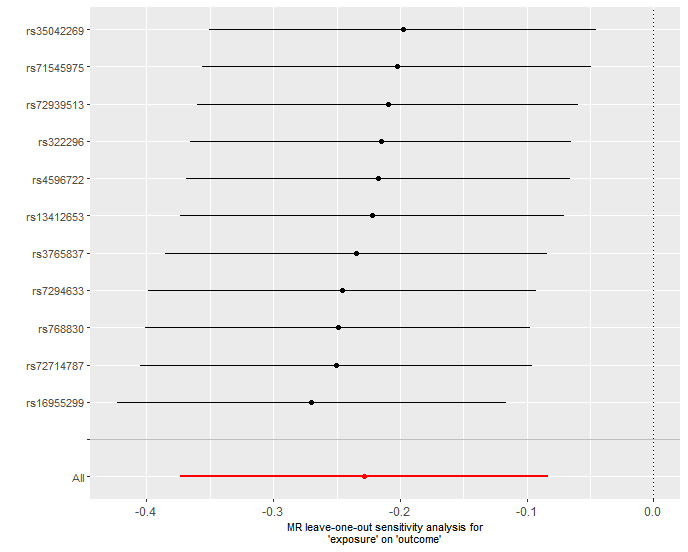

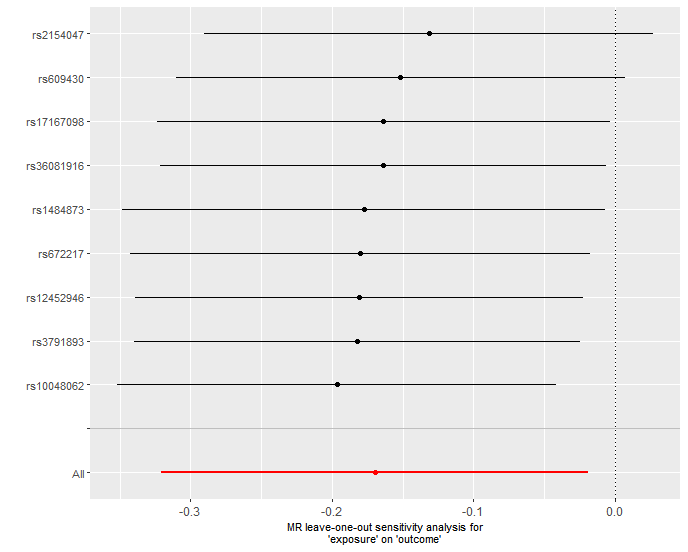

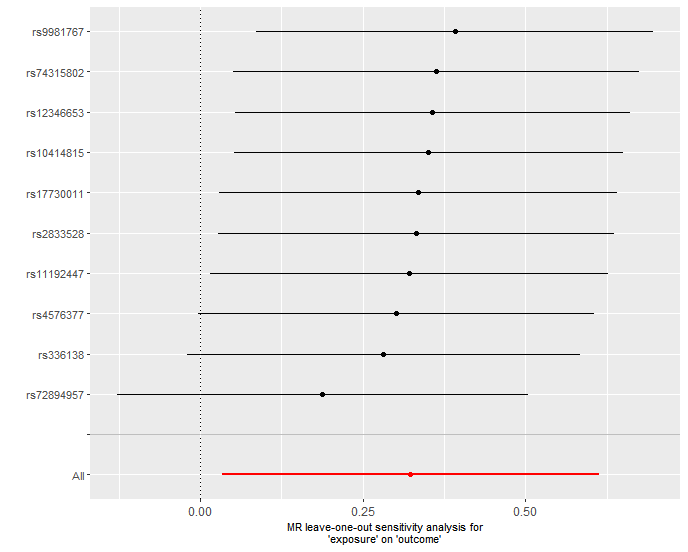

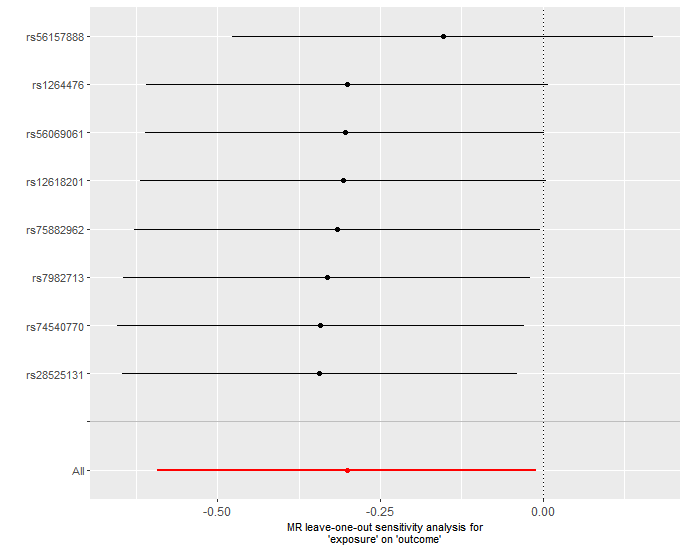

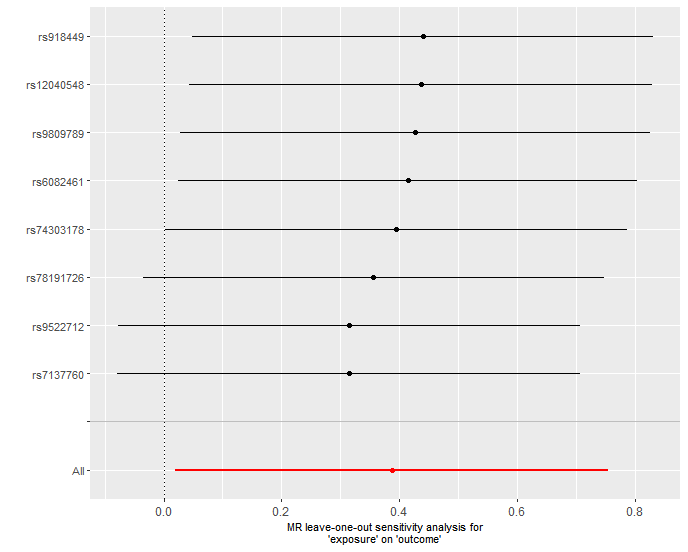

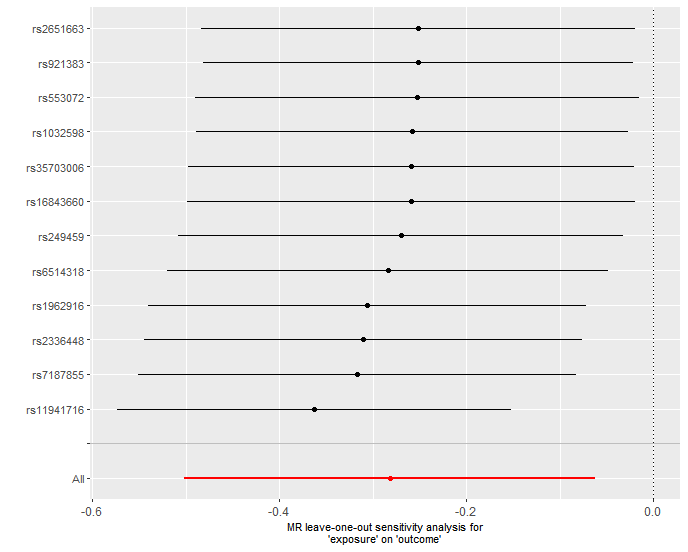


**A**

**B**

**C**

**D**

**E**

**F**

**G**

**H**

**I**

**J**

**K**

**Supplementary Figure S5**

Leave-one-out plots of significant and nominal significant estimates from genetically predicted gut microbiota { (A) Class.Gammaproteobacteria.id.3303; (B) Family.ClostridialesvadinBB60group.id.11286; (C) Family.Rikenellaceae.id.967; (D) Genus.Anaerostipes.id.1991; (E) Genus.Anaerotruncus.id.2054; (F) Genus.Coprococcus3.id.11303; (G) Genus.Ruminiclostridium9.id.11357; (H) Genus.RuminococcaceaeUCG011.id.11368; (I) Genus.unknowngenus.id.2041; (J) Genus.unknowngenus.id.1000000073; (K) Phylum.Cyanobacteria.id.1500 } on D-C/R.


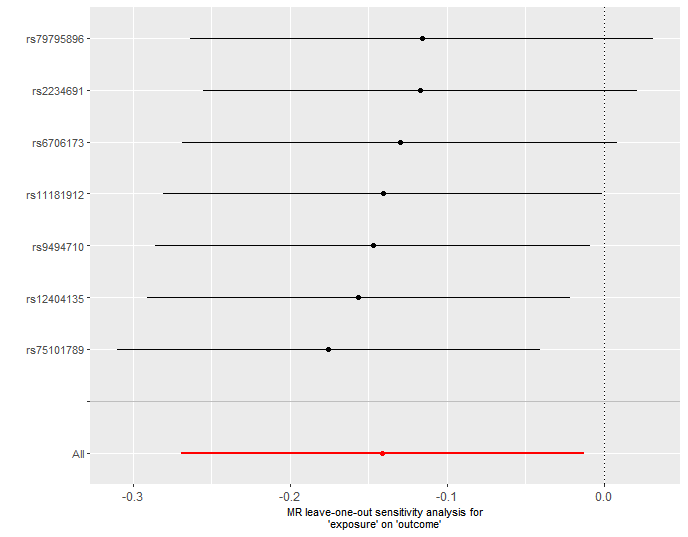

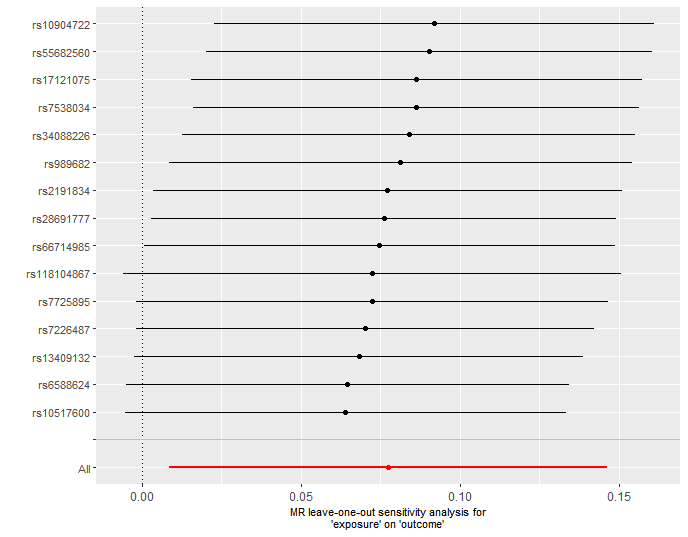

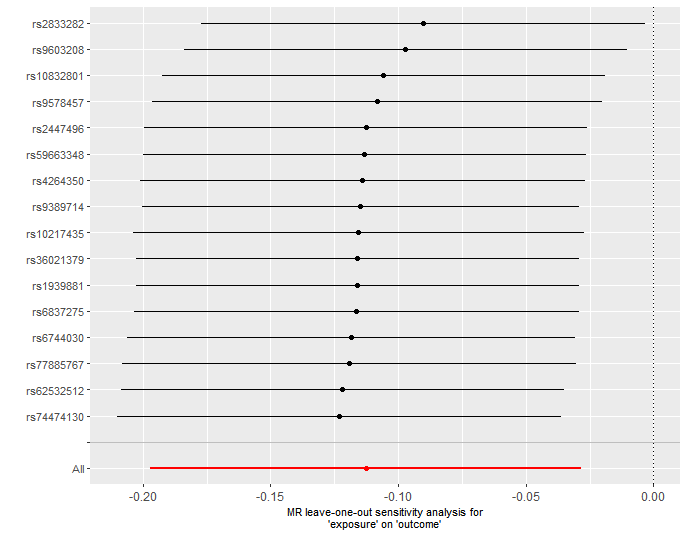

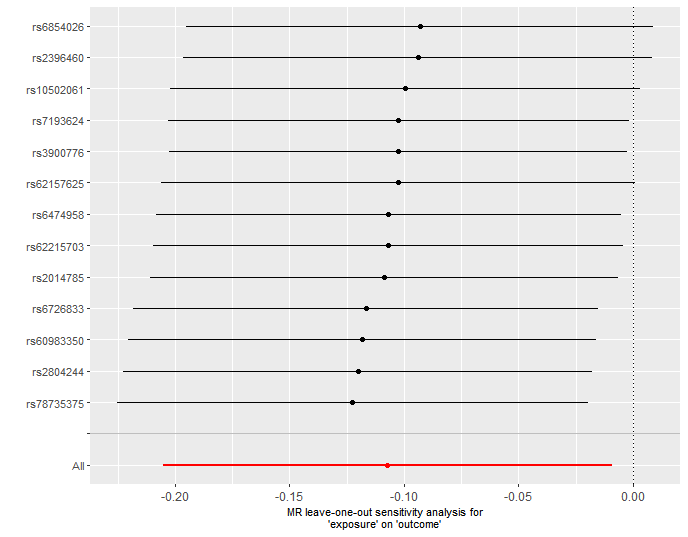

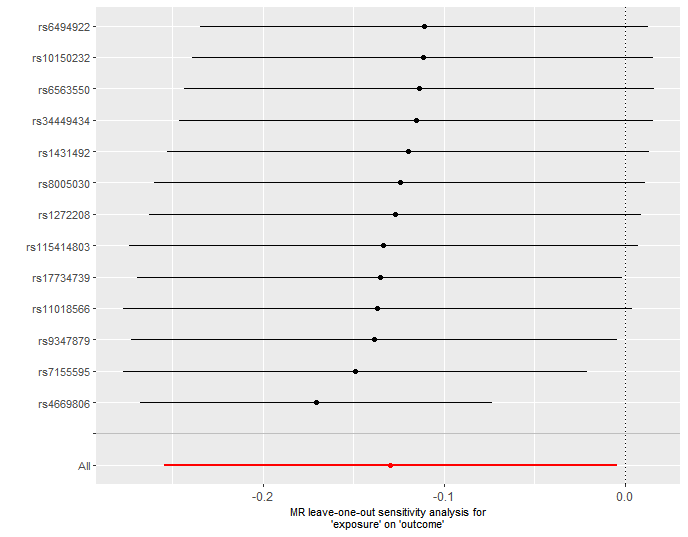

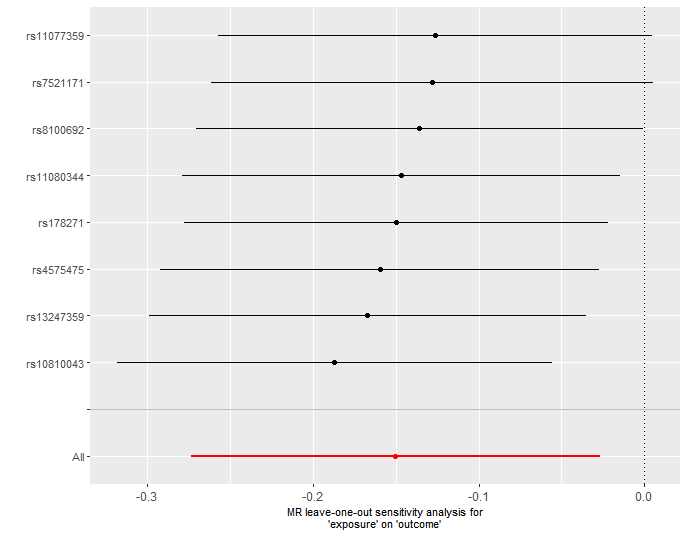

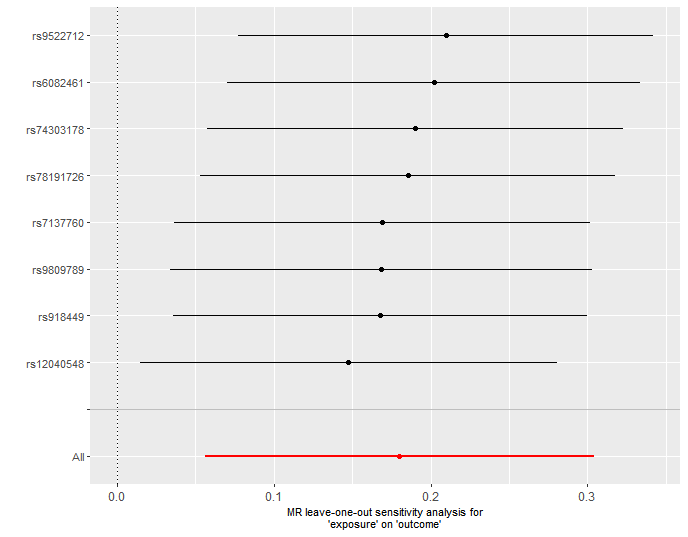

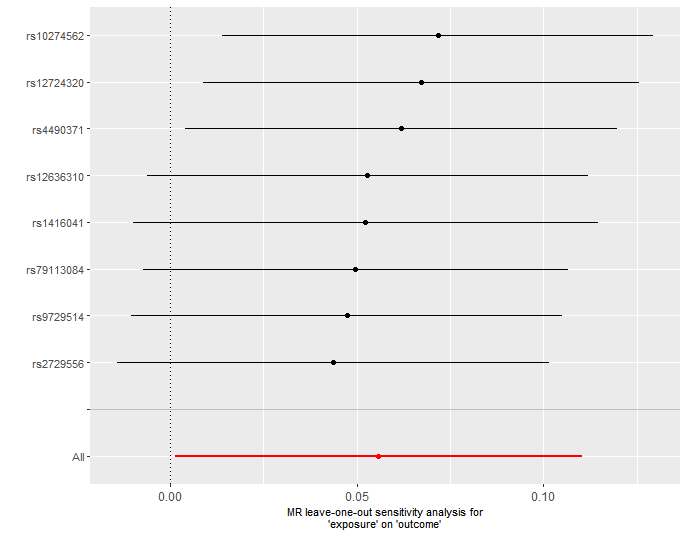

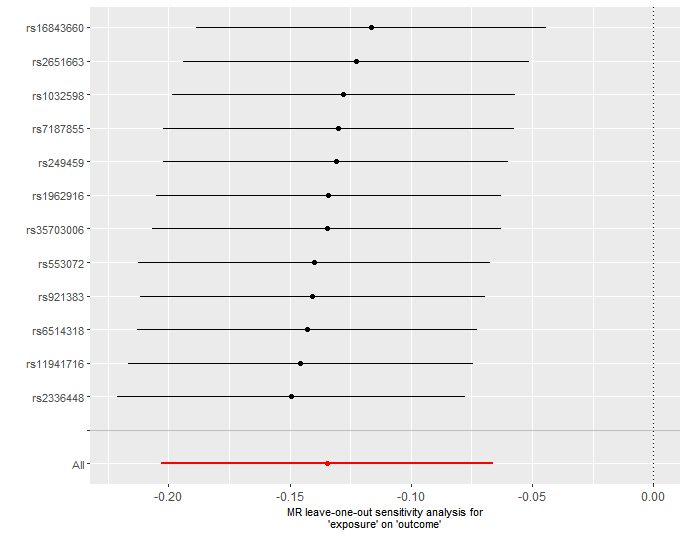

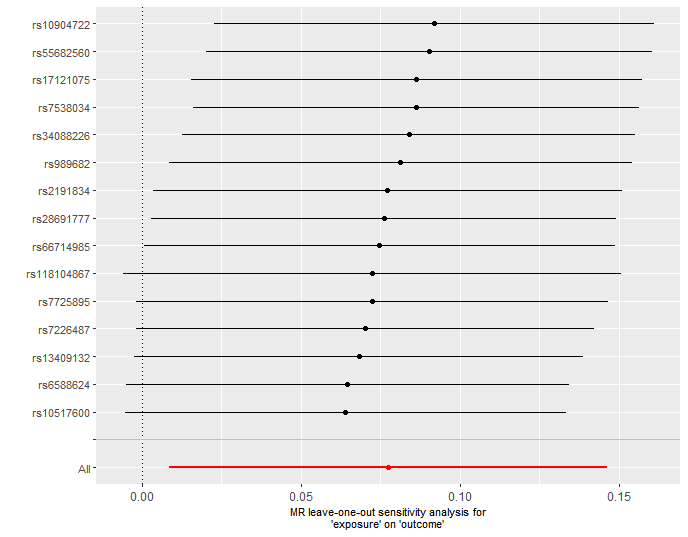

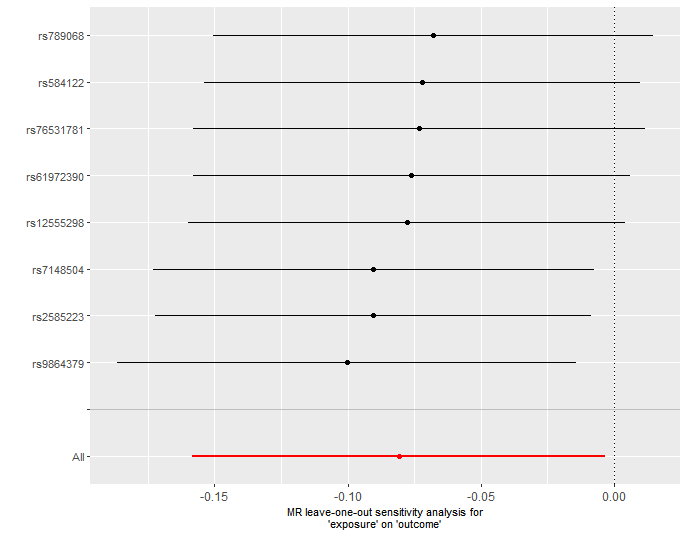


**A**

**B**

**C**

**D**

**E**

**F**

**G**

**H**

**I**

**J**

**K**

**Supplementary Figure S6**

Leave-one-out plots of significant and nominal significant estimates from genetically predicted gut microbiota { (A) Family.BacteroidalesS24.7group.id.11173; (B) Family.Oxalobacteraceae.id.2966; (C) Genus.Oxalobacter.id.2978; (D) Genus.Parasutterella.id.2892; (E) Genus.unknowngenus.id.1000005479; (F) Phylum.Verrucomicrobia.id.3982 } on Visual Impairment.


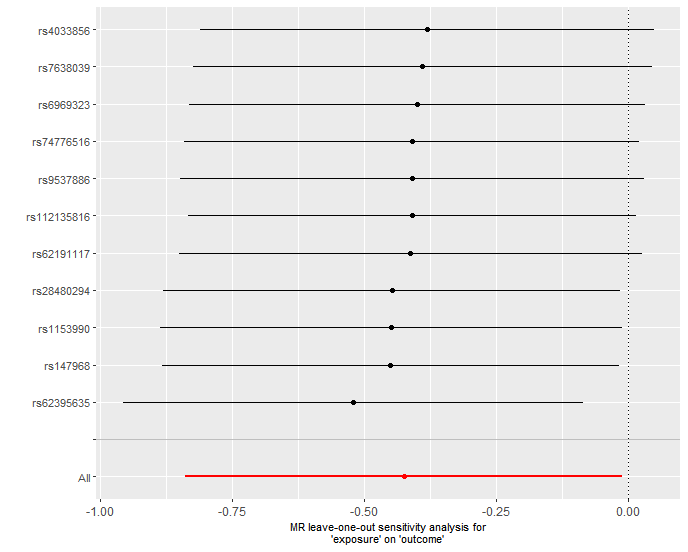

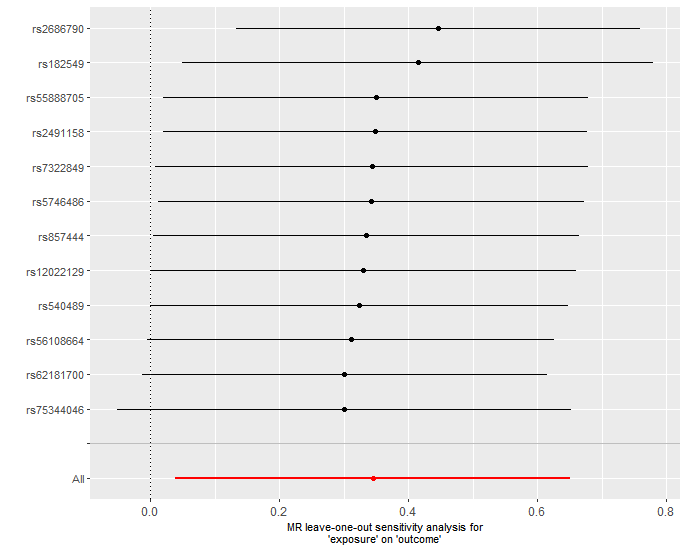


**A**

**B**

**Supplementary Figure S7**

Scatter plots of significant and nominal significant estimates from genetically predicted gut microbiota { (A) Class.Bacteroidia.id.912; (B) Family.ClostridialesvadinBB60group.id.11286; (C) Genus..Eubacteriumeligensgroup.id.14372; (D) Genus.Dialister.id.2183; (E) Genus.Gordonibacter.id.821; (F) Genus.RuminococcaceaeUCG003.id.11361; (G) Genus.unknowngenus.id.1868; (H) Genus.unknowngenus.id.2041; (I) Genus.unknowngenus.id.1000000073; (J) Order.Bacteroidales.id.913 } on DR.


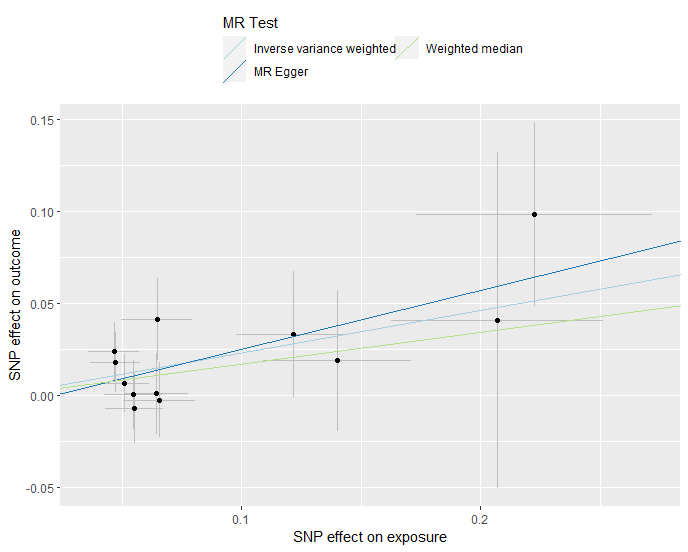

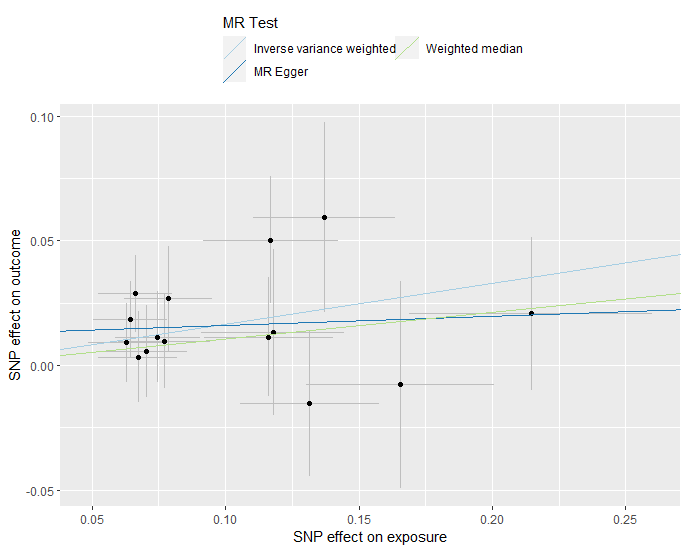

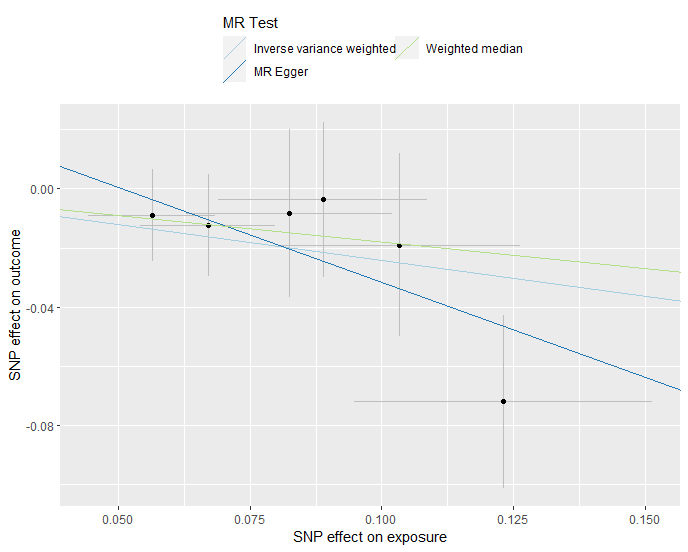

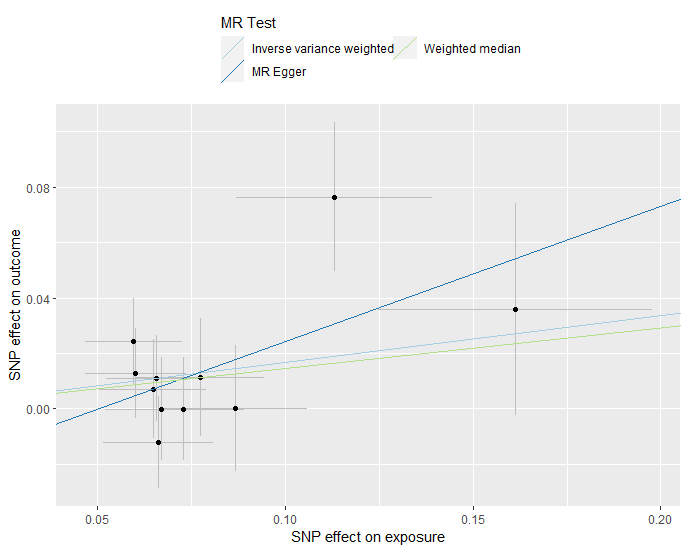

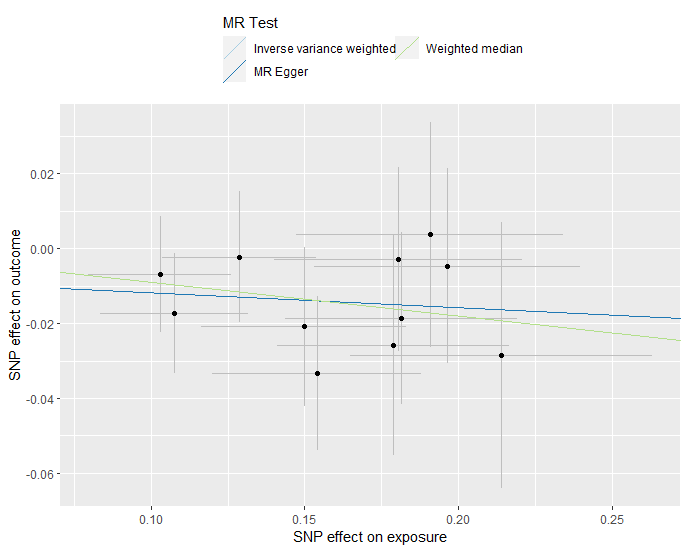

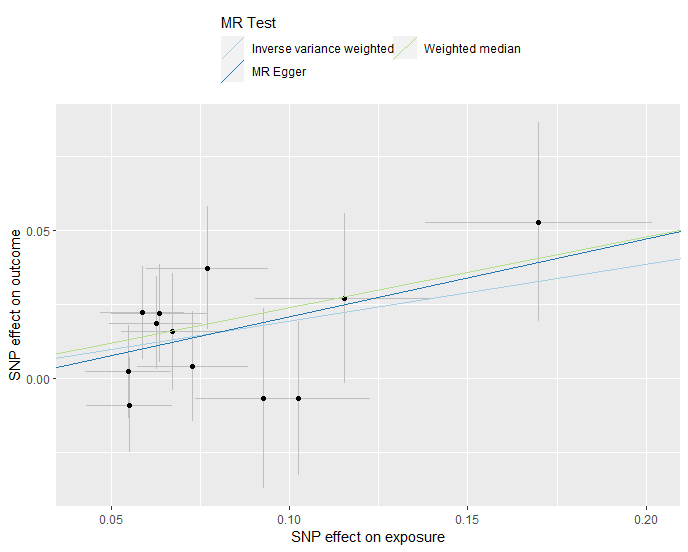

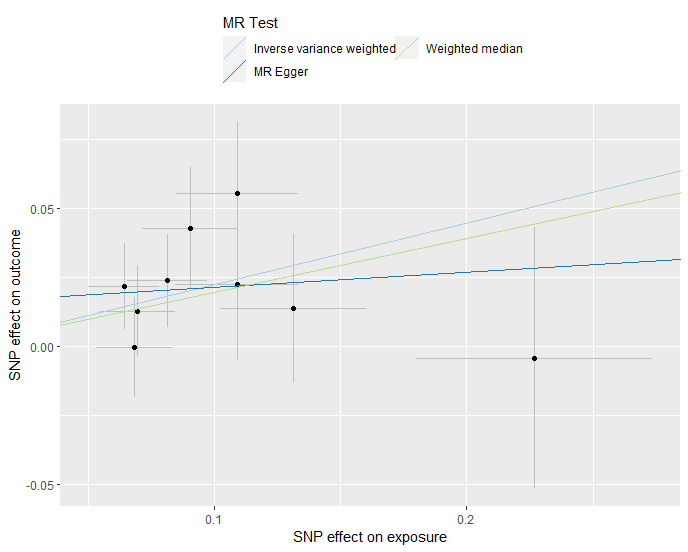

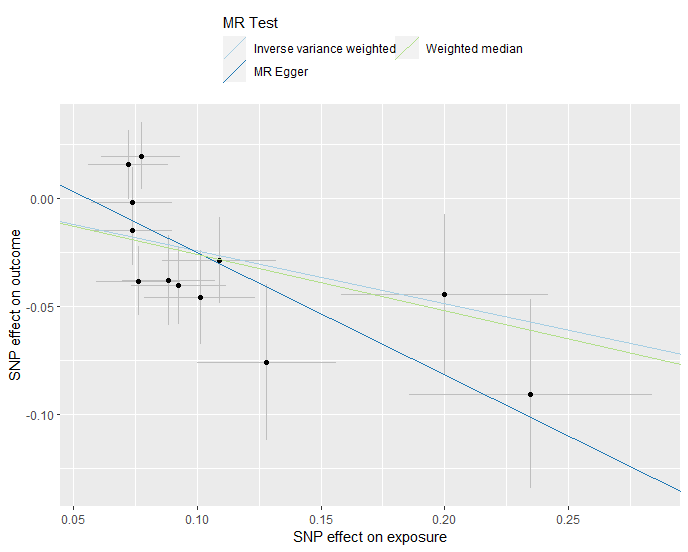

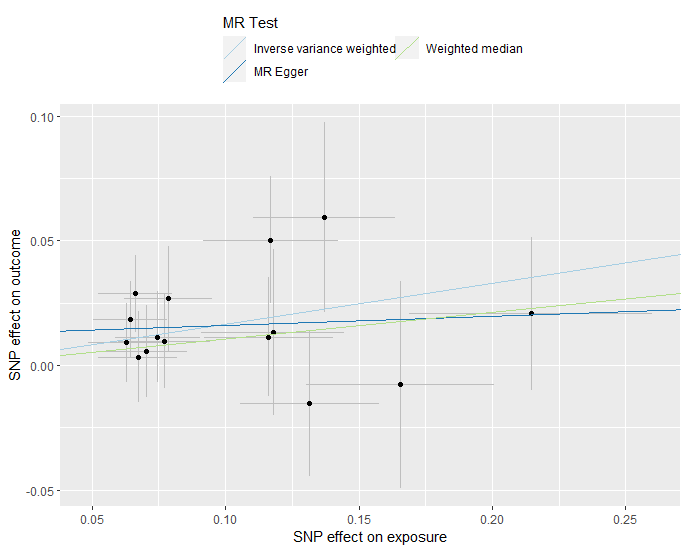

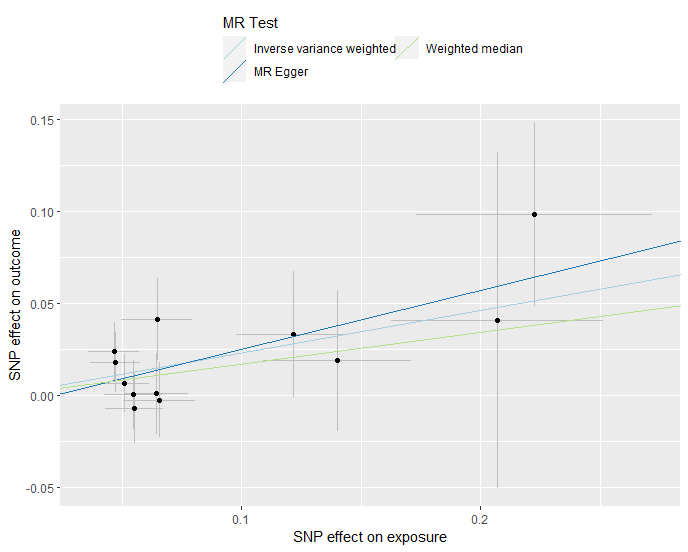


**A**

**B**

**C**

**D**

**E**

**F**

**G**

**H**

**I**

**J**

**Supplementary Figure S8**

Scatter plots of significant and nominal significant estimates from genetically predicted gut microbiota { (A) Family.BacteroidalesS24.7group.id.11173; (B) Family.Oxalobacteraceae.id.2966; (C) Genus.Oxalobacter.id.2978; (D) Genus.Parasutterella.id.2892; (E) Genus.unknowngenus.id.1000005479; (F) Phylum.Verrucomicrobia.id.3982 } on Eamd.


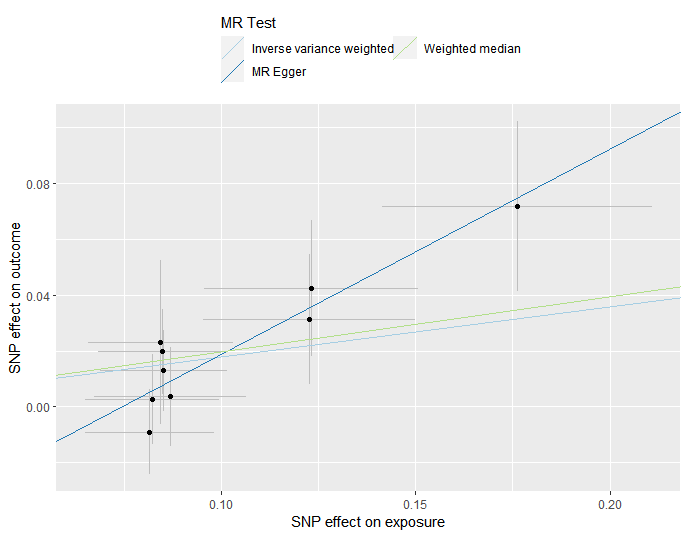

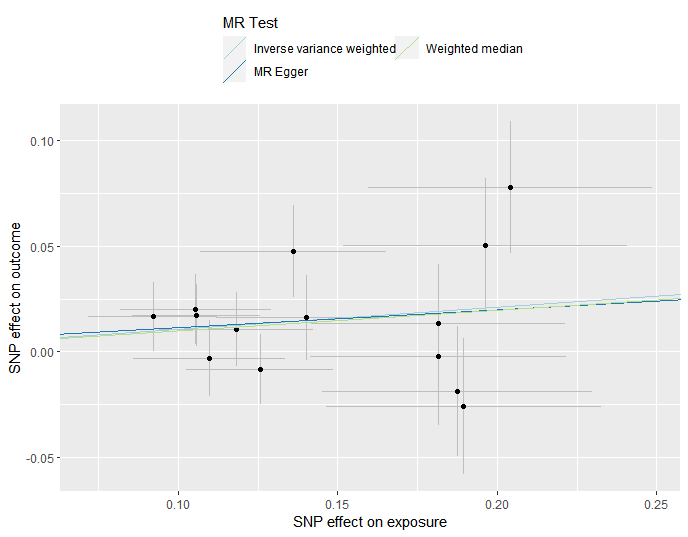

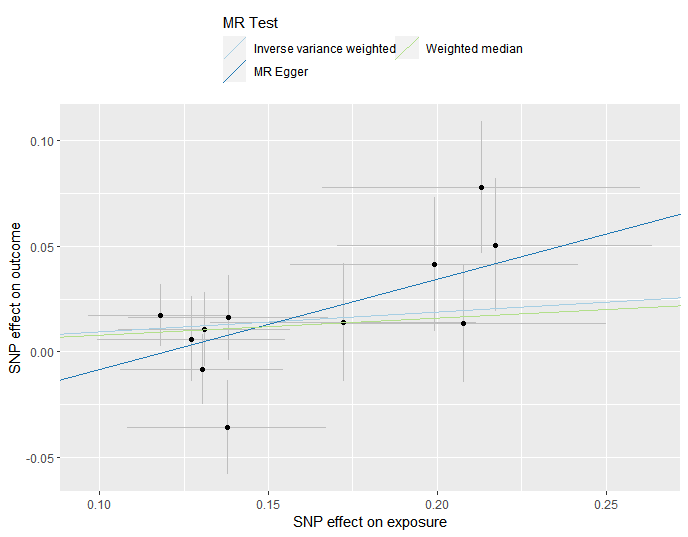

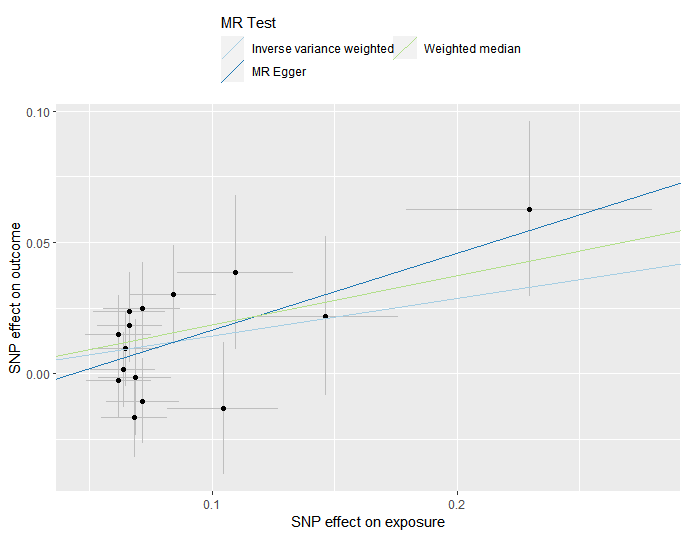

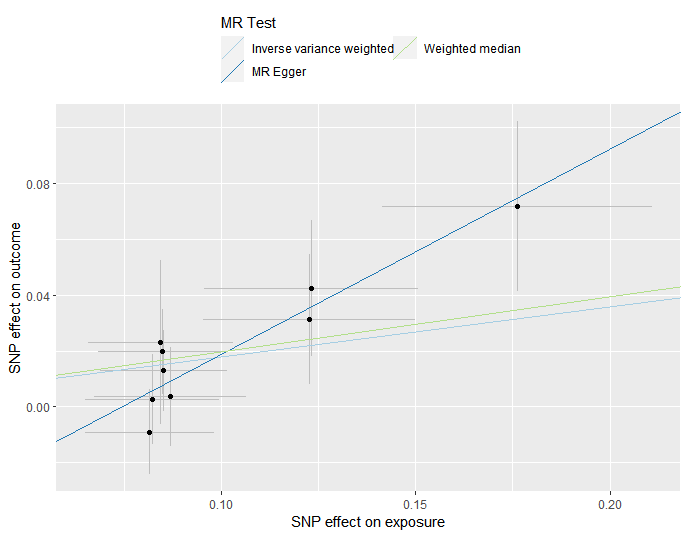

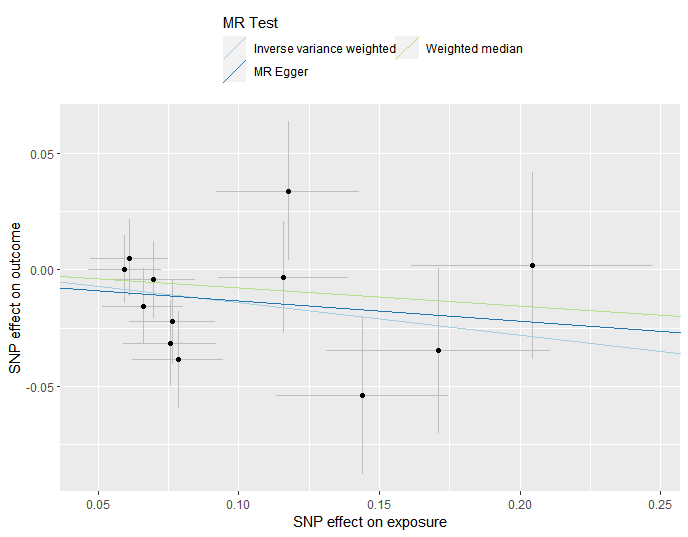


**A**

**B**

**C**

**D**

**E**

**F**

**Supplementary Figure S9**

Scatter plots of significant and nominal significant estimates from genetically predicted gut microbiota { (A) Genus.Flavonifractor.id.2059; (B) Genus.Intestinibacter.id.11345; (C) Genus.Ruminiclostridium9.id.11357; (D) Genus.unknowngenus.id.2041 } on RD/RB.


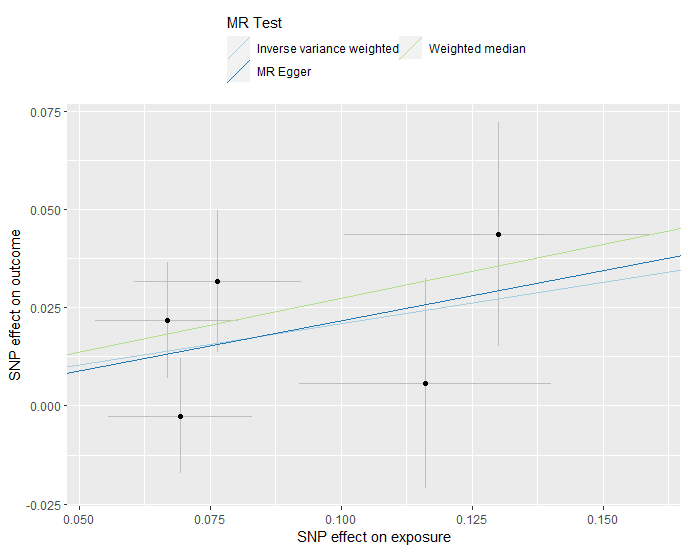

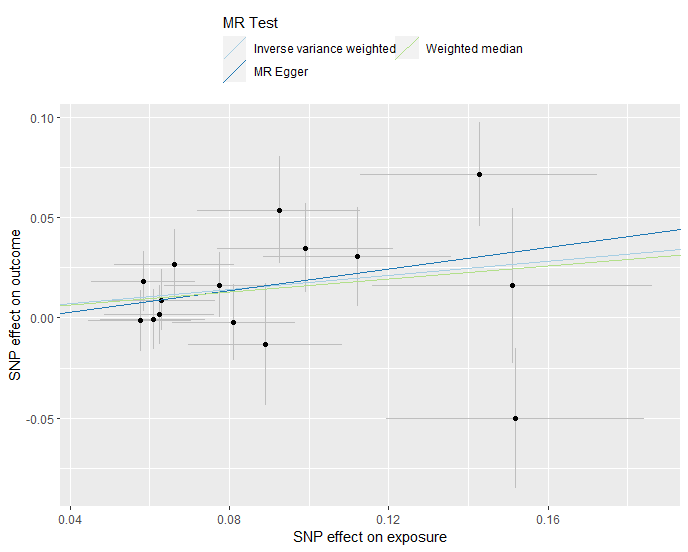

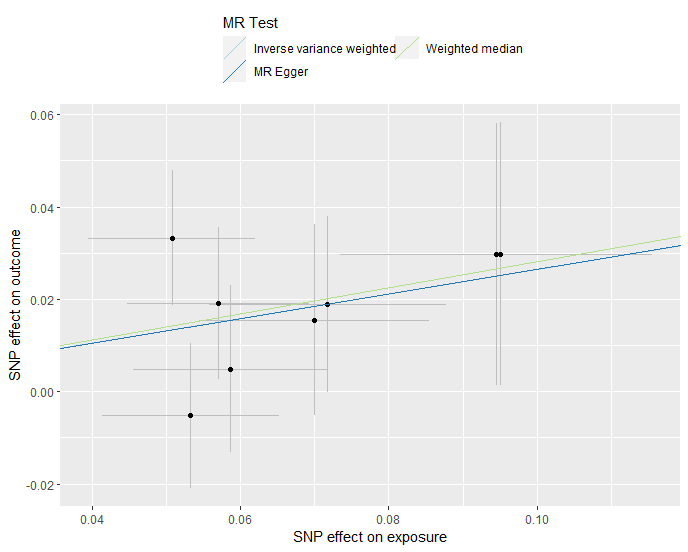

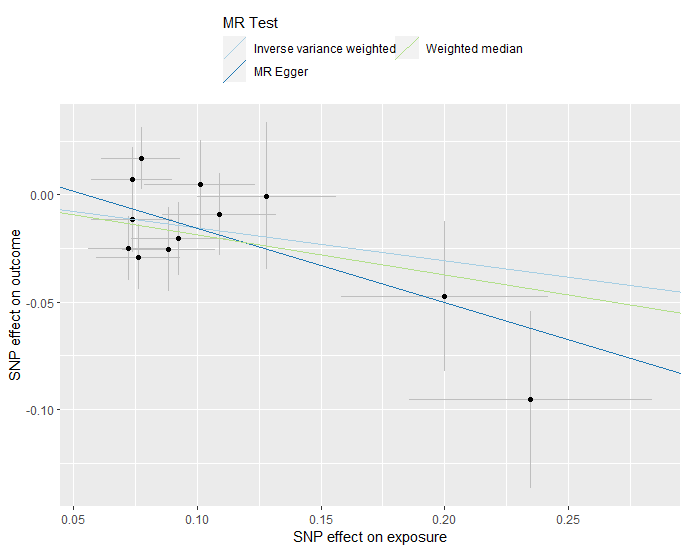


**A**

**B**

**C**

**D**

**Supplementary Figure S10**

Scatter plots of significant and nominal significant estimates from genetically predicted gut microbiota { (A) Genus..Ruminococcusgnavusgroup.id.14376; (B) Genus.Anaerotruncus.id.2054; (C)Genus.Butyricicoccus.id.2055; (D) Genus.Butyricimonas.id.945; (E) Genus.Clostridiumsensustricto1.id.1873; (F) Genus.Gordonibacter.id.821; (G) Genus.Howardella.id.2000; (H) Genus.LachnospiraceaeUCG010.id.11330; (I) Genus.Phascolarctobacterium.id.2168; (J) Genus.Ruminiclostridium9.id.11357 ; (K) Genus.unknowngenus.id.2041 } on RVO.


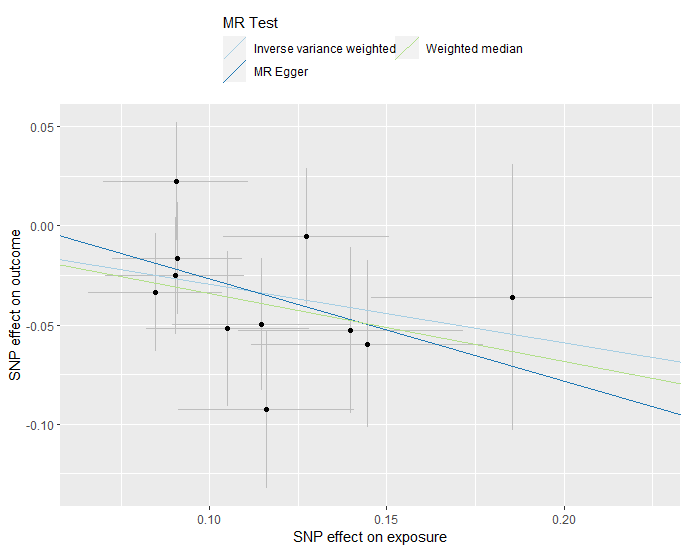

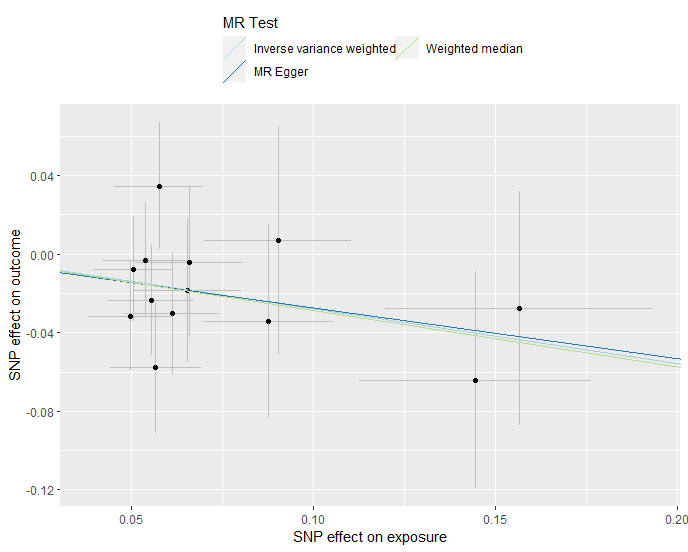

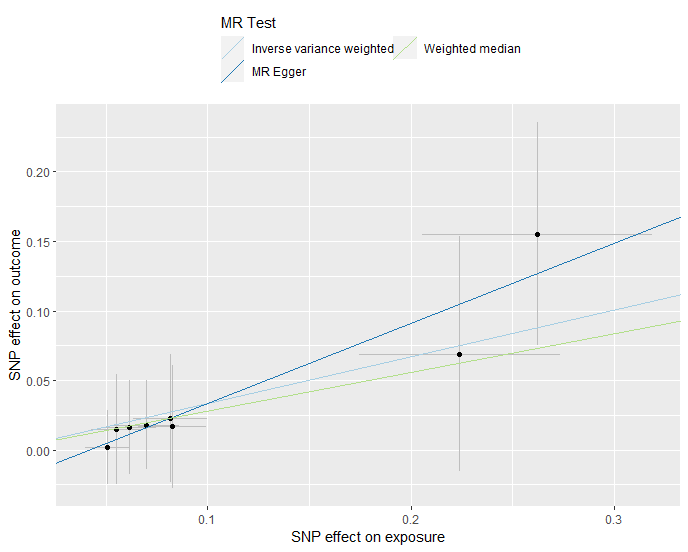

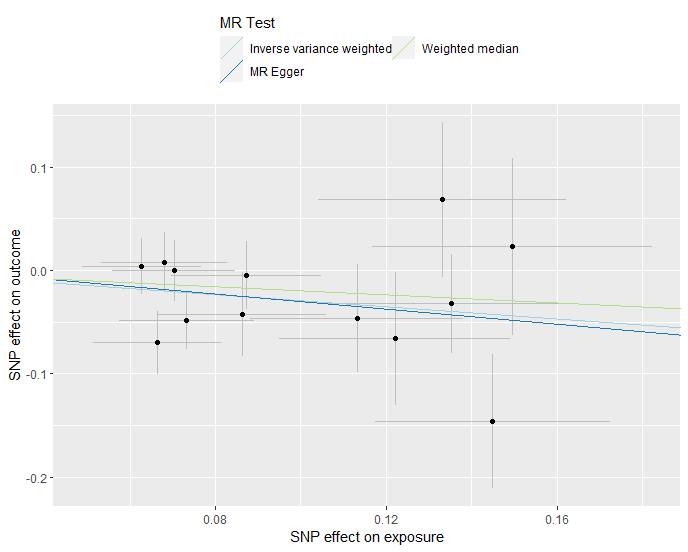

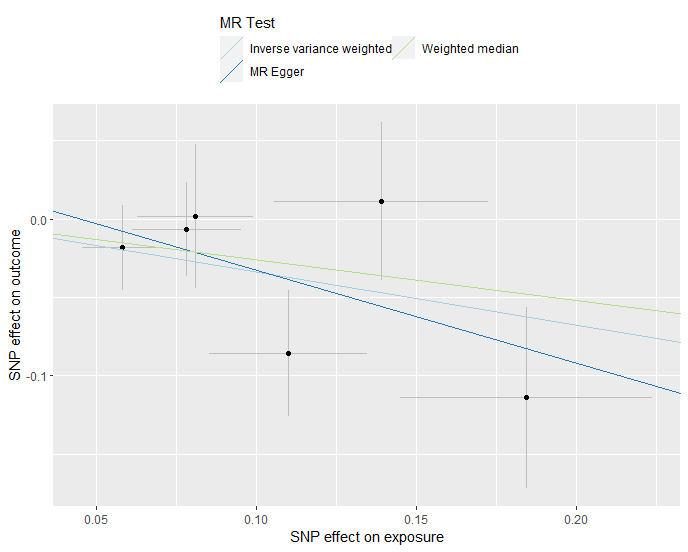

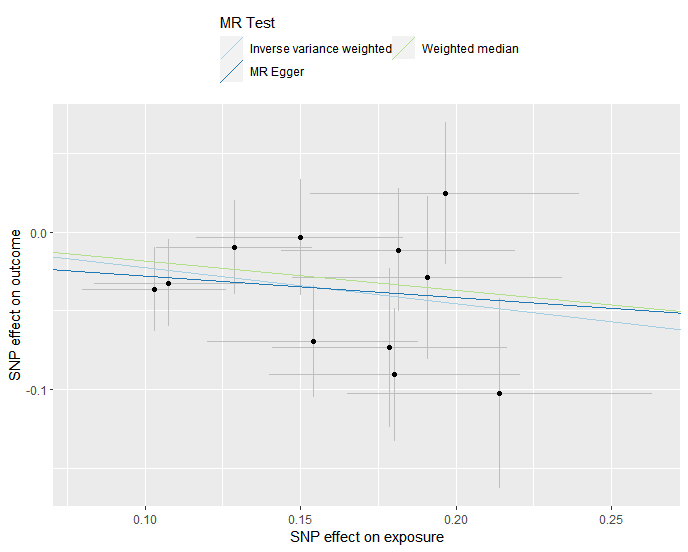

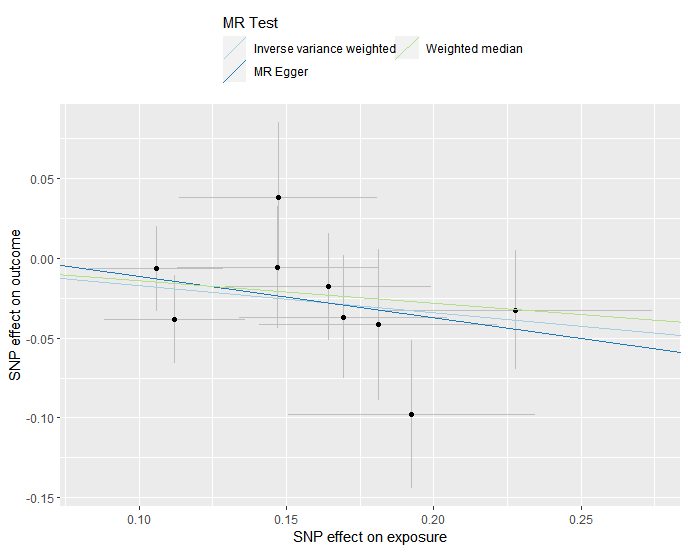

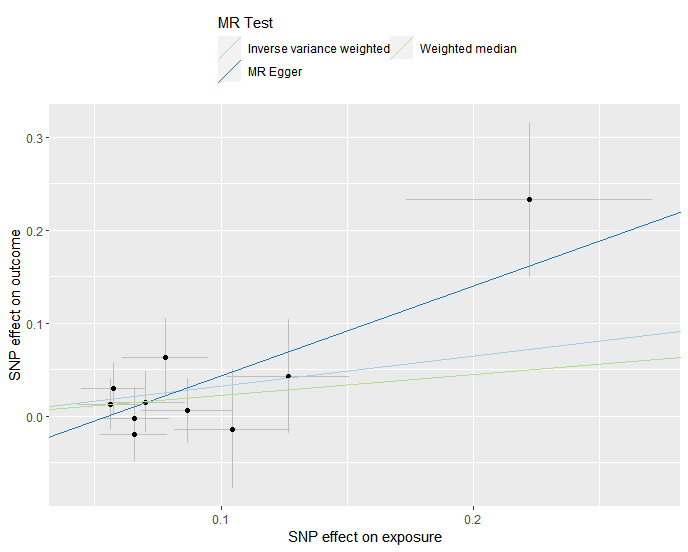

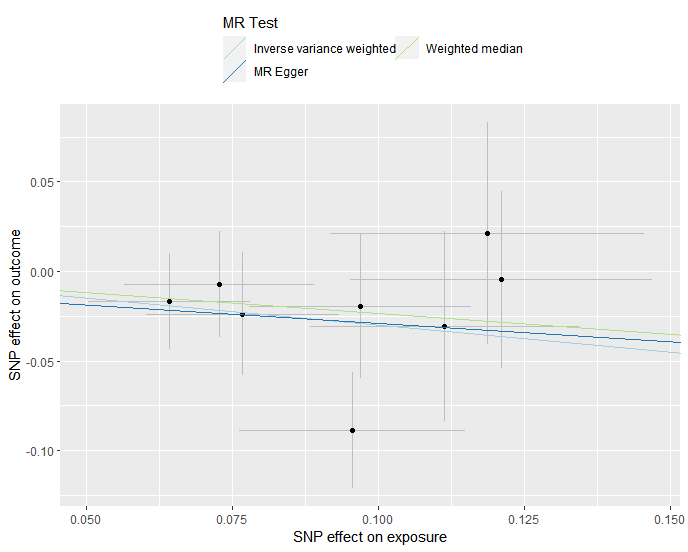

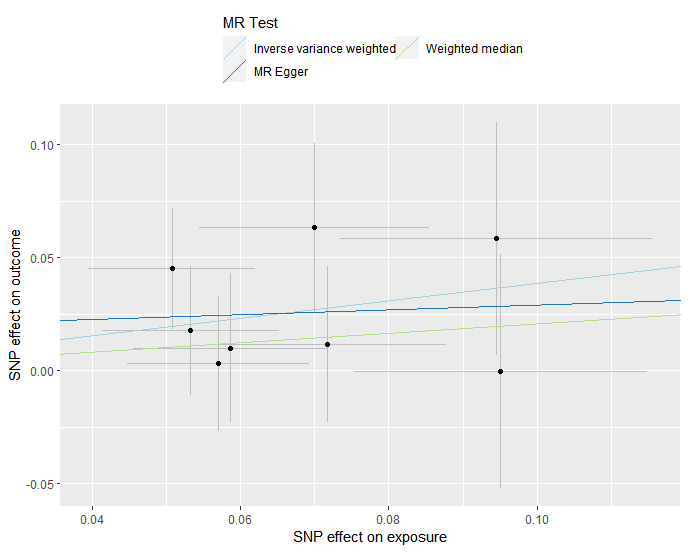

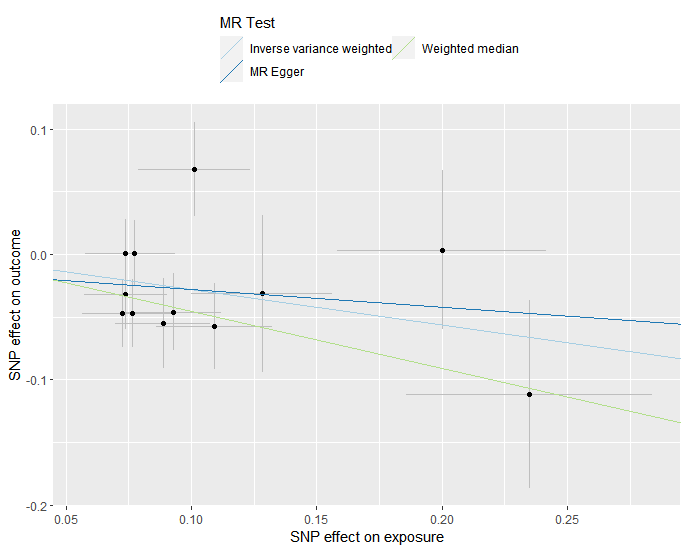


**A**

**B**

**C**

**D**

**E**

**F**

**G**

**H**

**I**

**J**

**K**

**Supplementary Figure S11**

Scatter plots of significant and nominal significant estimates from genetically predicted gut microbiota { (A) Class.Gammaproteobacteria.id.3303; (B) Family.ClostridialesvadinBB60group.id.11286; (C) Family.Rikenellaceae.id.967; (D) Genus.Anaerostipes.id.1991; (E) Genus.Anaerotruncus.id.2054; (F) Genus.Coprococcus3.id.11303; (G) Genus.Ruminiclostridium9.id.11357; (H) Genus.RuminococcaceaeUCG011.id.11368; (I) Genus.unknowngenus.id.2041; (J) Genus.unknowngenus.id.1000000073; (K) Phylum.Cyanobacteria.id.1500 } on D-C/R.


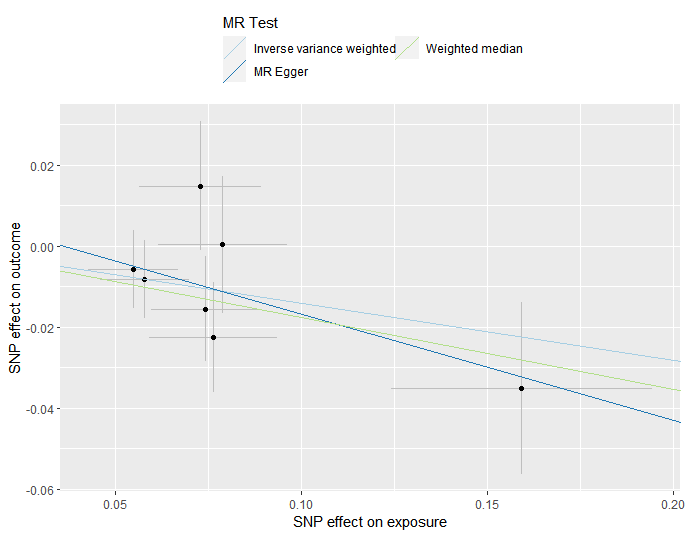

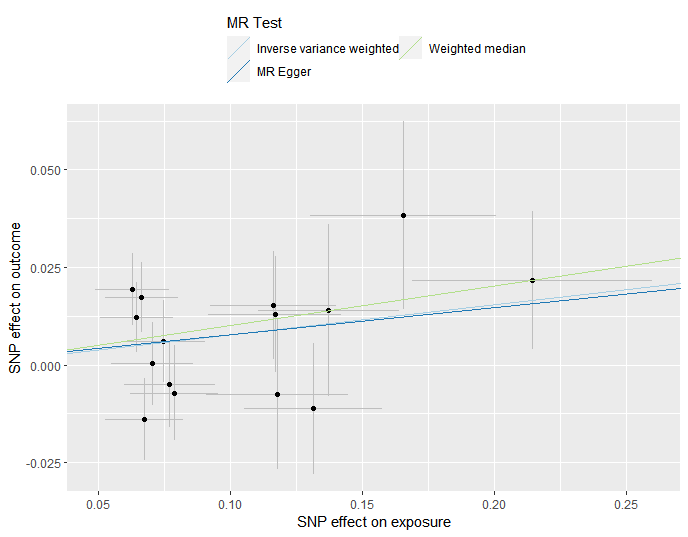

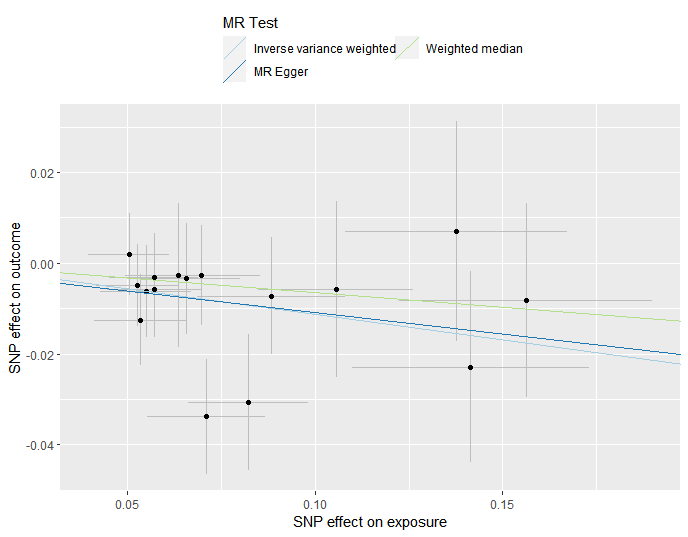

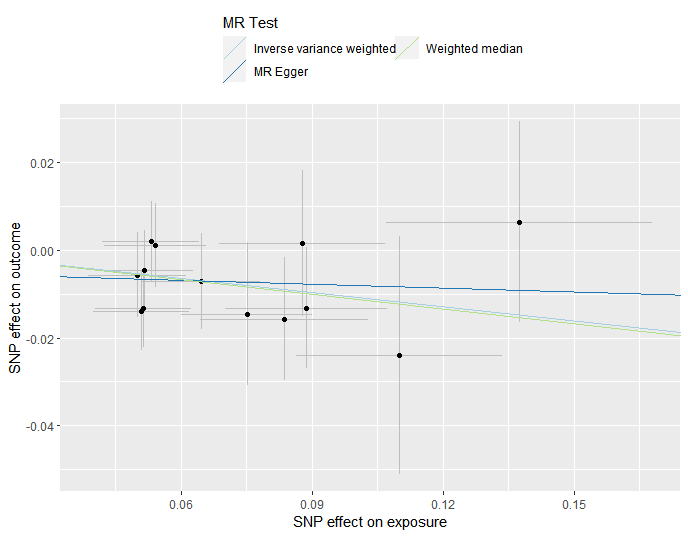

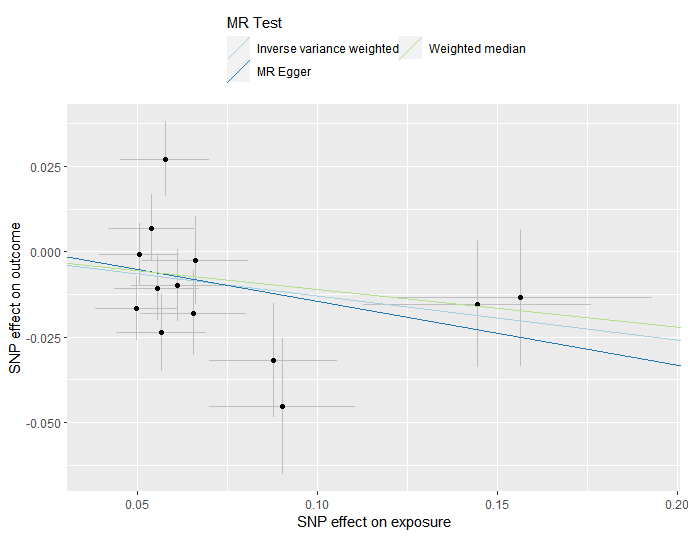

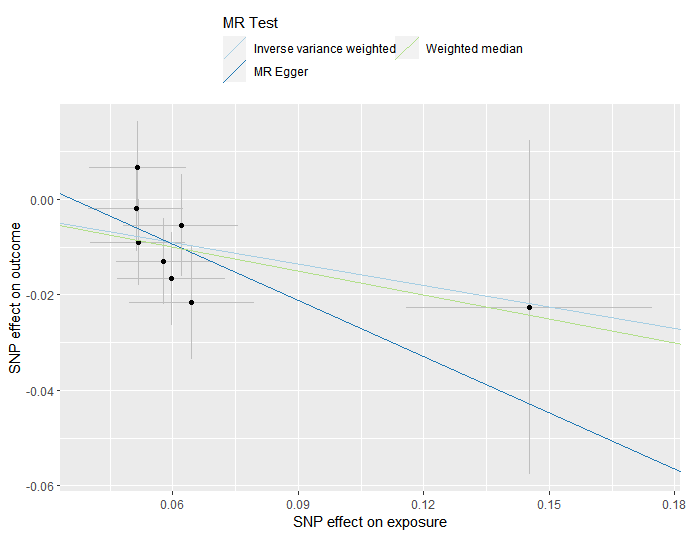

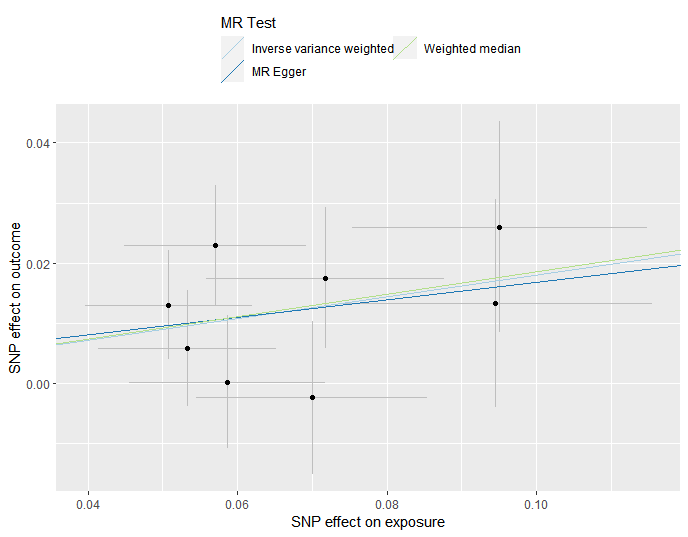

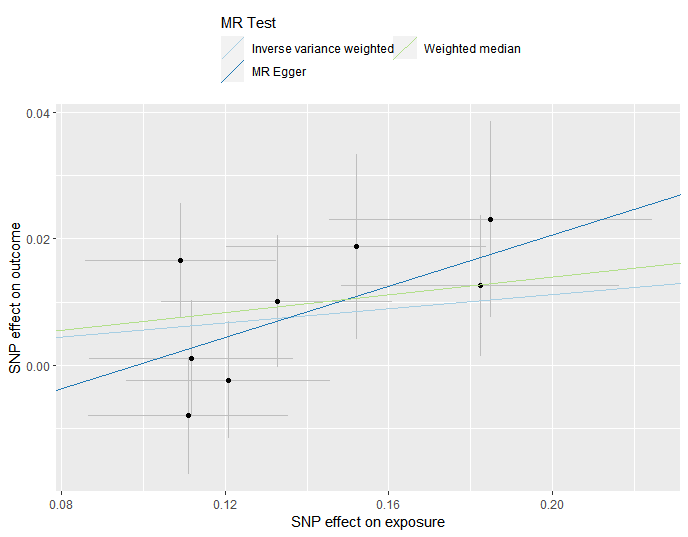

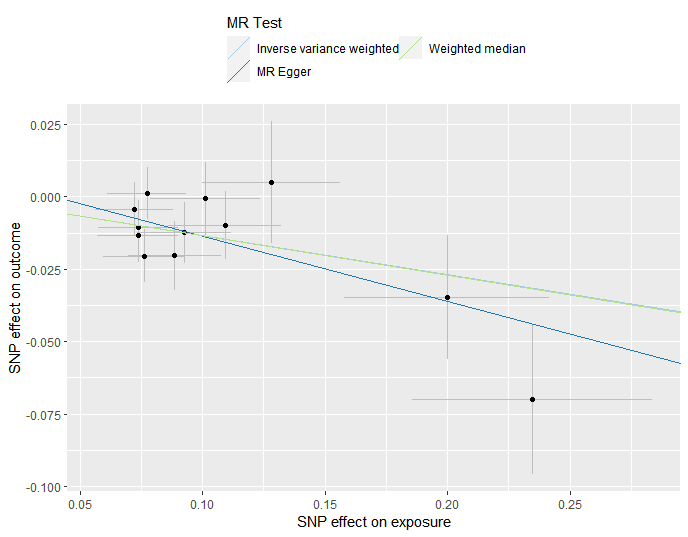

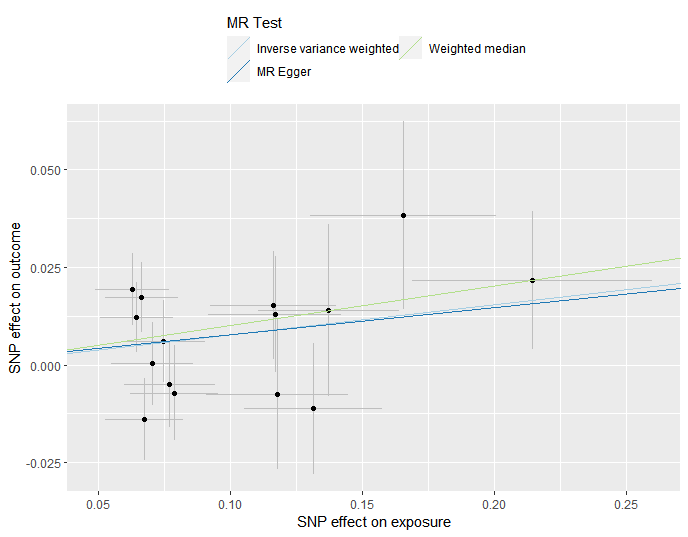

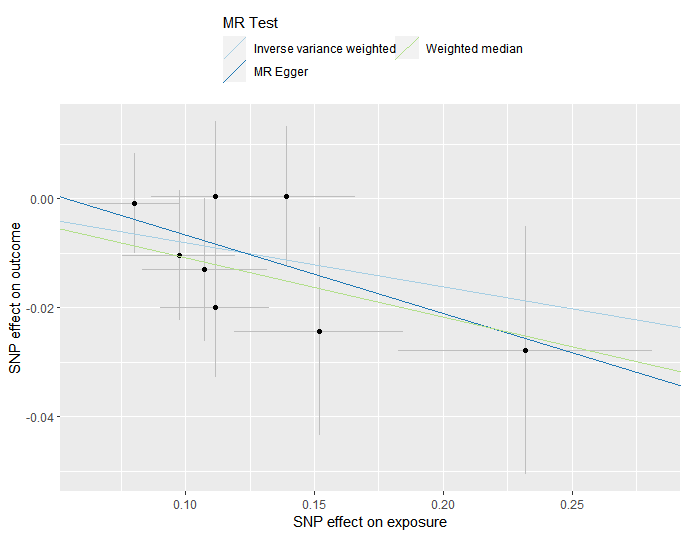


**A**

**B**

**C**

**D**

**E**

**F**

**G**

**H**

**I**

**J**

**K**

**Supplementary Figure S12**

Scatter plots of significant and nominal significant estimates from genetically predicted gut microbiota { (A) Family.BacteroidalesS24.7group.id.11173; (B) Family.Oxalobacteraceae.id.2966; (C) Genus.Oxalobacter.id.2978; (D) Genus.Parasutterella.id.2892; (E) Genus.unknowngenus.id.1000005479; (F) Phylum.Verrucomicrobia.id.3982 } on Visual Impairment.


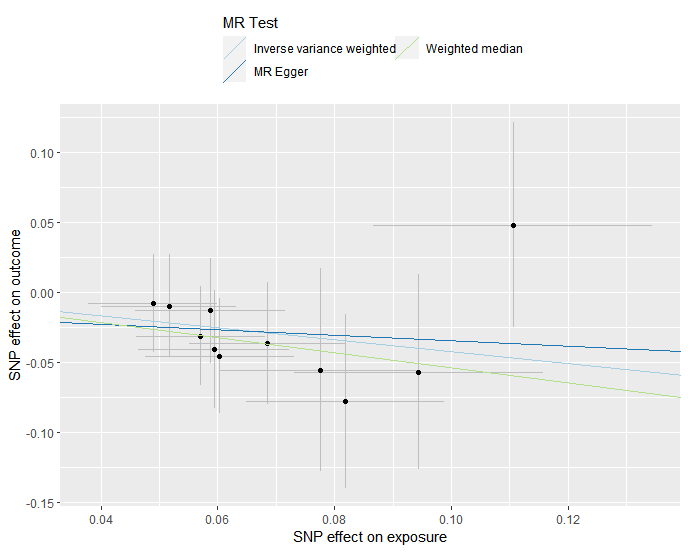

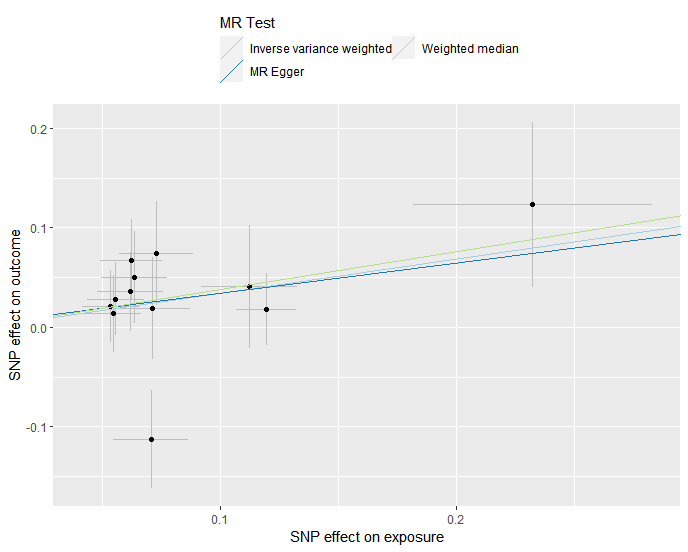


**A**

**B**

**Supplementary Figure S13**

Funnel plots of significant and nominal significant estimates from genetically predicted gut microbiota { (A) Class.Bacteroidia.id.912; (B) Family.ClostridialesvadinBB60group.id.11286; (C) Genus..Eubacteriumeligensgroup.id.14372; (D) Genus.Dialister.id.2183; (E) Genus.Gordonibacter.id.821; (F) Genus.RuminococcaceaeUCG003.id.11361; (G) Genus.unknowngenus.id.1868; (H) Genus.unknowngenus.id.2041; (I) Genus.unknowngenus.id.1000000073; (J) Order.Bacteroidales.id.913 } on DR.


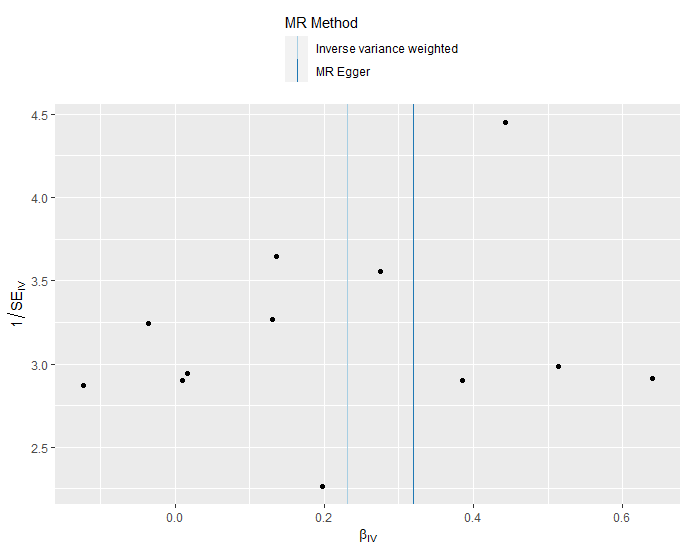

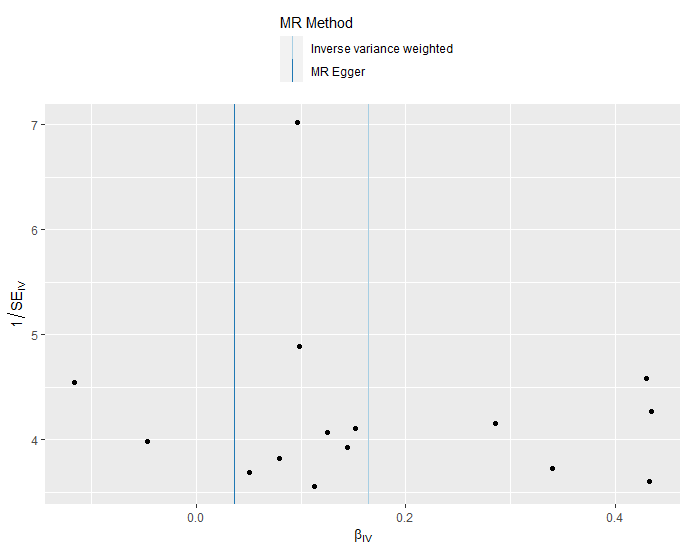

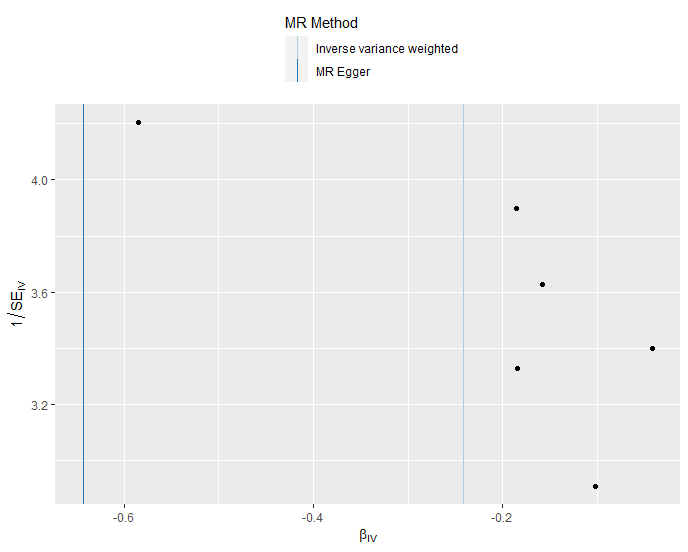

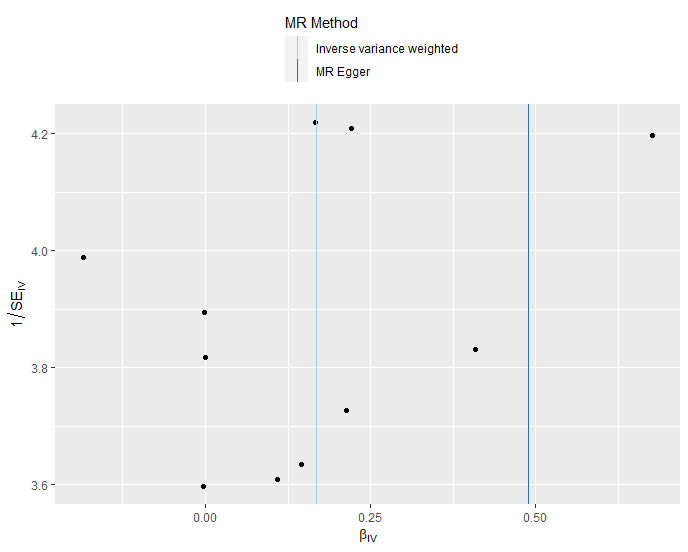

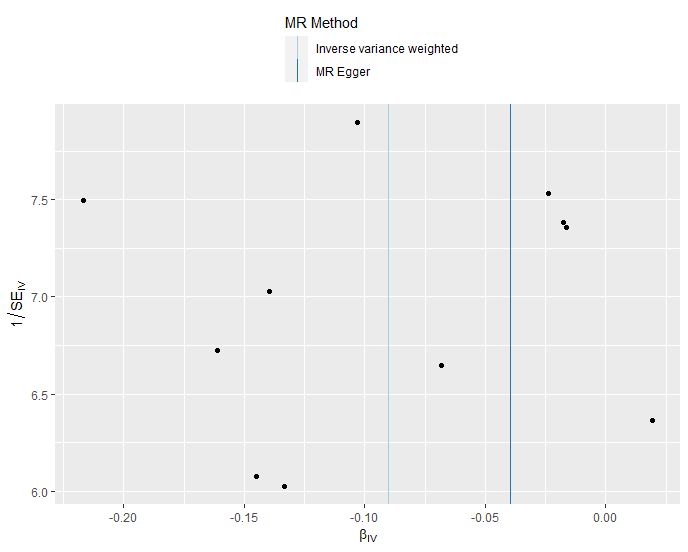

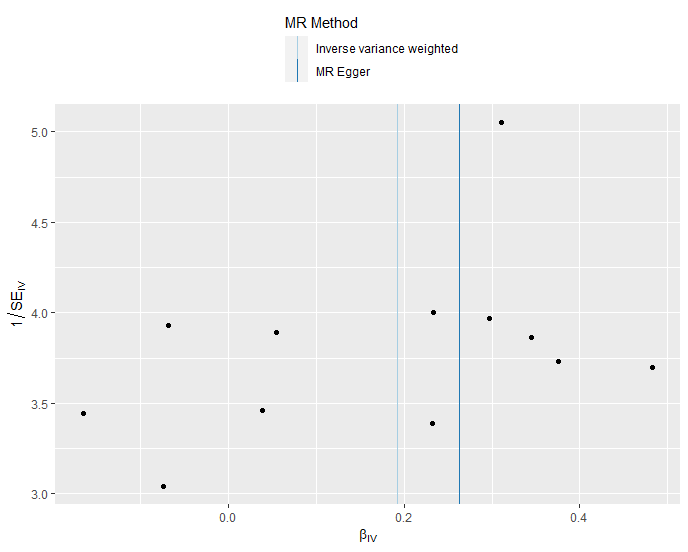

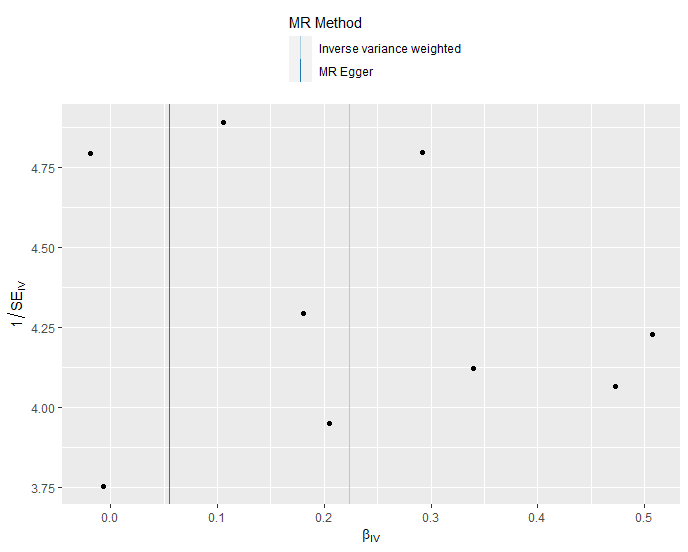

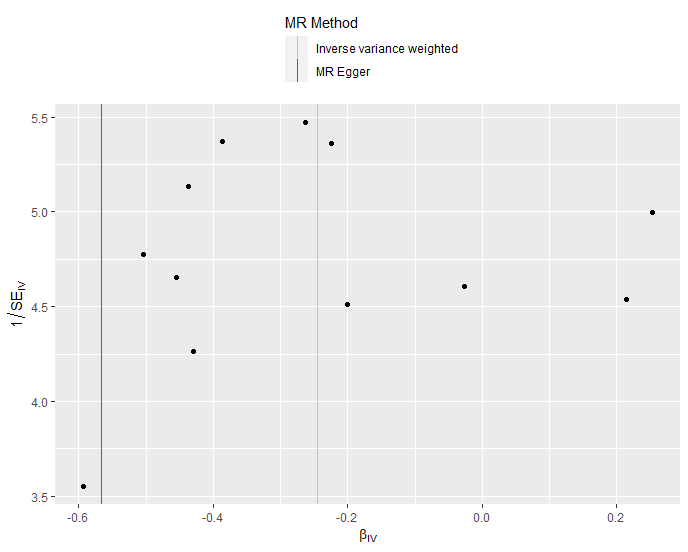

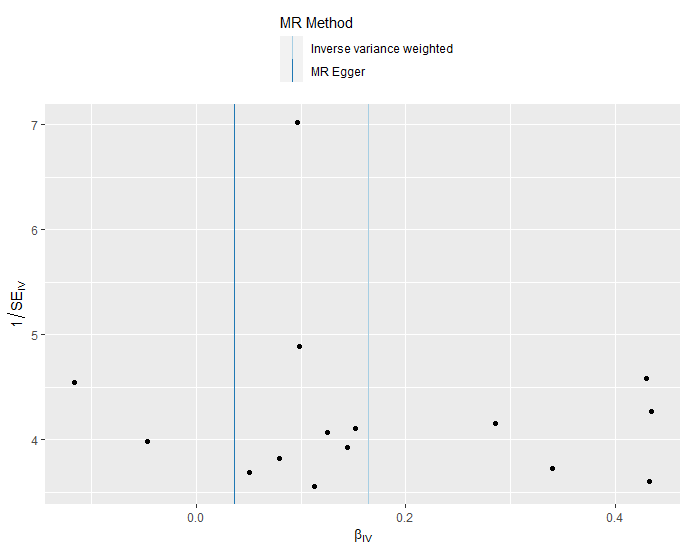

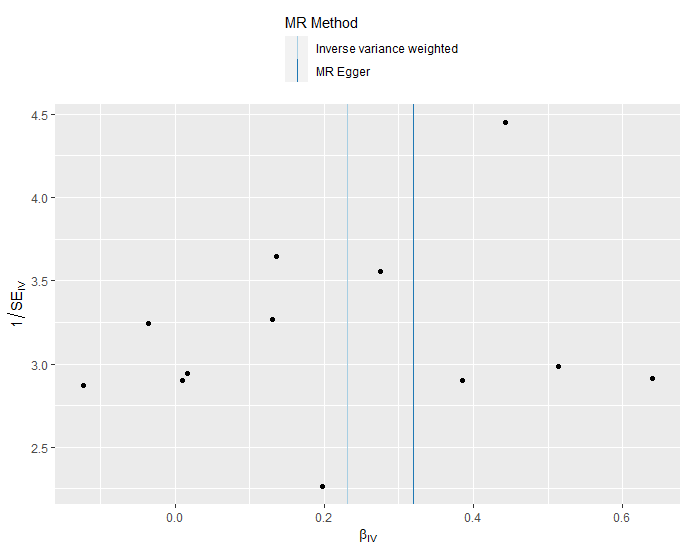


**A**

**B**

**C**

**D**

**E**

**F**

**G**

**H**

**I**

**J**

**Supplementary Figure S14**

Funnel plots of significant and nominal significant estimates from genetically predicted gut microbiota { (A) Family.BacteroidalesS24.7group.id.11173; (B) Family.Oxalobacteraceae.id.2966; (C) Genus.Oxalobacter.id.2978; (D) Genus.Parasutterella.id.2892; (E) Genus.unknowngenus.id.1000005479; (F) Phylum.Verrucomicrobia.id.3982 } on eAMD.


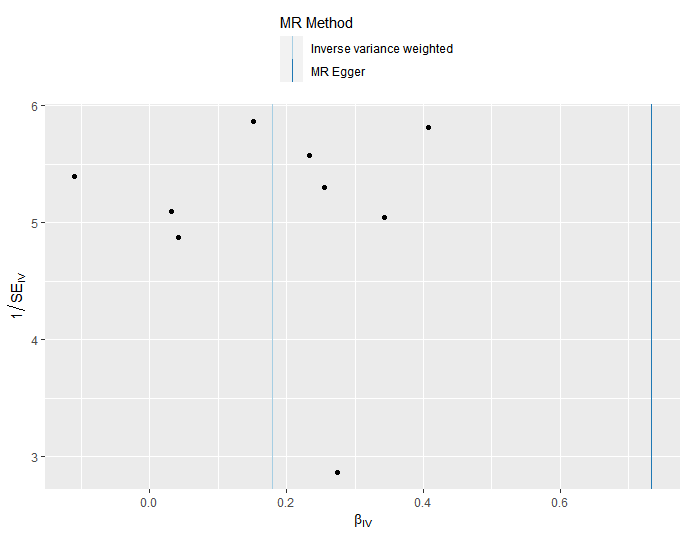

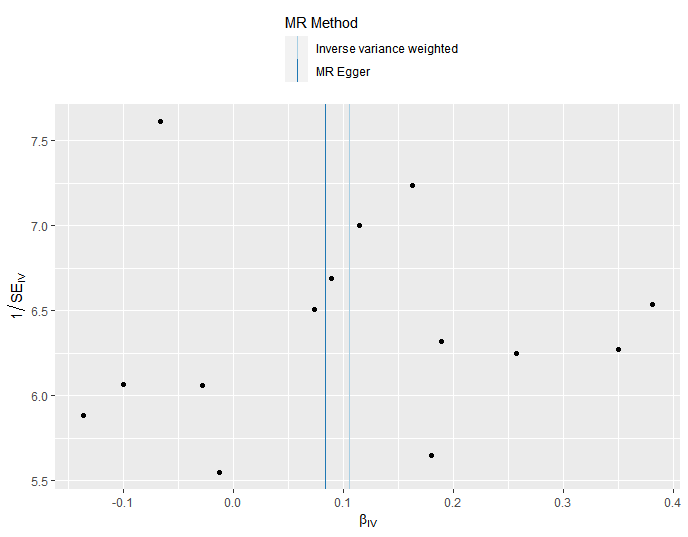


**A**

**B**

**C**

**D**

**E**

**F**

**Supplementary Figure S15**

Funnel plots of significant and nominal significant estimates from genetically predicted gut microbiota { (A) Genus.Flavonifractor.id.2059; (B) Genus.Intestinibacter.id.11345; (C) Genus.Ruminiclostridium9.id.11357; (D) Genus.unknowngenus.id.2041 } on RD/RB.

**A**

**B**

**C**

**D**

**Supplementary Figure S16**

Funnel plots of significant and nominal significant estimates from genetically predicted gut microbiota { (A) Genus..Ruminococcusgnavusgroup.id.14376; (B) Genus.Anaerotruncus.id.2054; (C)Genus.Butyricicoccus.id.2055; (D) Genus.Butyricimonas.id.945; (E) Genus.Clostridiumsensustricto1.id.1873; (F) Genus.Gordonibacter.id.821; (G) Genus.Howardella.id.2000; (H) Genus.LachnospiraceaeUCG010.id.11330; (I) Genus.Phascolarctobacterium.id.2168; (J) Genus.Ruminiclostridium9.id.11357 ; (K) Genus.unknowngenus.id.2041 } on RVO.

**A**

**B**

**C**

**D**

**E**

**F**

**G**

**H**

**I**

**J**

**K**

**Supplementary Figure S17**

Funnel plots of significant and nominal significant estimates from genetically predicted gut microbiota { (A) Class.Gammaproteobacteria.id.3303; (B) Family.ClostridialesvadinBB60group.id.11286; (C) Family.Rikenellaceae.id.967; (D) Genus.Anaerostipes.id.1991; (E) Genus.Anaerotruncus.id.2054; (F) Genus.Coprococcus3.id.11303; (G) Genus.Ruminiclostridium9.id.11357; (H) Genus.RuminococcaceaeUCG011.id.11368; (I) Genus.unknowngenus.id.2041; (J) Genus.unknowngenus.id.1000000073; (K) Phylum.Cyanobacteria.id.1500 } on D-C/R.

**A**

**B**

**C**

**D**

**E**

**F**

**G**

**H**

**I**

**J**

**K**

**Supplementary Figure S18**

Funnel plots of significant and nominal significant estimates from genetically predicted gut microbiota { (A) Family.BacteroidalesS24.7group.id.11173; (B) Family.Oxalobacteraceae.id.2966; (C) Genus.Oxalobacter.id.2978; (D) Genus.Parasutterella.id.2892; (E) Genus.unknowngenus.id.1000005479; (F) Phylum.Verrucomicrobia.id.3982 } on Visual Impairment.

**A**

**B**
